# Morphological evolution of various fungal species in the presence and absence of aluminum oxide microparticles: Comparative and quantitative insights into microparticle‐enhanced cultivation (MPEC)

**DOI:** 10.1002/mbo3.603

**Published:** 2018-03-05

**Authors:** Anna Kowalska, Tomasz Boruta, Marcin Bizukojć

**Affiliations:** ^1^ Faculty of Process and Environmental Engineering Department of Bioprocess Engineering Lodz University of Technology Lodz Poland

**Keywords:** filamentous fungi, image analysis, microparticle‐enhanced cultivation, morphology engineering

## Abstract

The application of microparticle‐enhanced cultivation (MPEC) is an attractive method to control mycelial morphology, and thus enhance the production of metabolites and enzymes in the submerged cultivations of filamentous fungi. Unfortunately, most literature data deals with the spore‐agglomerating species like aspergilli. Therefore, the detailed quantitative study of the morphological evolution of four different fungal species (*Aspergillus terreus*,* Penicillium rubens*,* Chaetomium globosum*, and *Mucor racemosus*) based on the digital analysis of microscopic images was presented in this paper. In accordance with the current knowledge, these species exhibit different mechanisms of agglomerates formation. The standard submerged shake flask cultivations (as a reference) and MPEC involving 10 μm aluminum oxide microparticles (6 g·L^−1^) were performed. The morphological parameters, including mean projected area, elongation, roughness, and morphology number were determined for the mycelial objects within the first 24 hr of growth. It occurred that heretofore observed and widely discussed effect of microparticles on fungi, namely the decrease in pellet size, was not observed for the species whose pellet formation mechanism is different from spore agglomeration. In the MPEC,* C. globosum* developed core‐shell pellets, and *M. racemosus*, a nonagglomerative species, formed the relatively larger, compared to standard cultures, pellets with distinct cores.

## 1 INTRODUCTION

Filamentous fungi are of central relevance to biotechnological industry due to their prolific biosynthetic capabilities. Widely exploited for the production of valuable metabolites and enzymes, these organisms constitute the core of important large‐scale biomanufacturing processes (Adrio & Demain, 2003; Demain & Martens, 2017). The development of cost‐effective cultivation strategies is thus an essential prerequisite for harnessing the enormous biochemical potential of fungal strains. Furthermore, maintaining the economic feasibility of bio‐based production is inevitably associated with the development of novel process‐related methods that allow for the continuous improvements in terms of titer, yield, and productivity (Nielsen et al., 2017).

Several approaches have been devised to influence one of the key parameters associated with submerged cultivations of filamentous fungi, namely the morphology (Walisko, Moench‐Tegeder, Blotenberg, Wucherpfennig, & Krull, 2015). Briefly, depending on a number of factors, the mycelial development in stirred or shaken liquid cultures proceeds in the forms of dispersed hyphae, clumps, or pellets (Papagianni, 2004). The latter can be either micropellets or macropellets whose diameter often exceeds even 1 mm. In filamentous fungi, three distinct mechanisms of pellets formation are distinguished, namely spores agglomeration (e.g., in *Aspergillus*), mycelial agglomeration (e.g., in *Penicillium*), and nonagglomerative pellet growth (e.g., in *Zygomycetes*). Since the optimal production of a particular fungal metabolite or enzyme is typically correlated with a specific morphological form, the biosynthesis of a target molecule can be greatly enhanced by controlling the morphology of the employed strain. It was previously reported that adding mineral microparticles (e.g., talc or aluminum oxide) to cultivation media has a profound effect on mycelial morphology in submerged cultures, and depending on the microbial platform and the molecule of interest, it can lead to the significant increase in product concentration via generating a favorable morphological form (Antecka, Blatkiewicz, Bizukojć, & Ledakowicz, 2016; Coban, Demirci, & Turhan, 2015a, 2015b; Coban and Demirci, 2016; Driouch, Hänsch, Wucherpfennig, Krull, & Wittmann, 2012; Driouch, Roth, Dersch, & Wittmann, 2010; Driouch, Sommer, & Wittmann, 2010; Etschmann et al., 2015; Gao, Zeng, Yu, Dong, & Chen, 2014; Gonciarz & Bizukojć, 2014; Kaup, Ehrich, Pescheck, & Schrader, 2008; Yatmaz, Karahalil, Germec, Ilgin, & Turhan, 2016). The effectiveness of this flexible, simple, and relatively inexpensive approach, referred to as the microparticle‐enhanced cultivation (MPEC), has been documented by several groups. For instance, the application of talc microparticles resulted in a dispersed morphology of *Aspergillus niger* SKAn 1015 and, consequently, a eightfold increase in β‐fructofuranosidase productivity was observed in fed‐batch bioreactor cultures (Driouch, Roth, et al. 2010). A similar phenomenon was noted for the fungus *Caldariomyces fumago* DSM 1256 by Kaup et al. (2008), who recorded a 10‐fold improvement in terms of chloroperoxidase formation in the talc‐enriched medium. Although the aforementioned studies involved the use of the MPEC for loosening the mycelia and elevating enzyme levels, Gonciarz and Bizukojć (2014) demonstrated the use of talc for decreasing pellet diameter and increasing product titers in the lovastatin‐oriented cultivation of *Aspergillus terreus* ATCC 20542. Other examples of MPEC‐related studies were reviewed by Antecka, Bizukojć, and Ledakowicz (2016), Krull et al. (2013), and Walisko et al. (2015). Currently, the MPEC is regarded as one of the leading modern methods of morphological engineering, a discipline defined by McIntyre, Müller, Dynesen, and Nielsen (2001) as “tailoring morphologies for specific bioprocesses.” In a broad perspective, the significant rise of metabolite or enzyme concentration in the MPEC proceeds in parallel with the change in substrate consumption rates, broth viscosity, and oxygen transfer (Antecka, Bizukojć, et al. 2016; Antecka, Blatkiewicz, et al. 2016).

Despite its advantages and confirmed effectiveness, the mechanism responsible for the interaction between microparticles and the growing mycelia has not been elucidated. Moreover, no quantitative representation of the MPEC‐related chain of events has been proposed so far. To the best of our knowledge, the only contribution in this respect was provided by Driouch, Sommer, et al. (2010), who verbally described the influence of talc microparticles on the agglomeration of *A. niger* SKAn1015 conidiospores in the submerged culture. According to those authors, the agglomeration is disturbed by microparticles in the initial phase of growth and, as a result, the pellets of reduced size or loose mycelia can be formed in the course of the cultivation (Driouch, Sommer, et al. 2010). Importantly, the morphology‐related measurements essential for the quantitative considerations were not performed. In the previously published works, the influence of the MPEC on fungal morphology was mainly analyzed in the context of an individually selected species, whereas a more comprehensive perspective is still lacking. Furthermore, the MPEC was mostly applied toward spore‐agglomerating aspergilli. Only Kaup et al. (2008) addressed the impact of microparticles on various filamentous microorganisms; however, the presented MPEC‐related dataset was rather limited in scope and referred solely to pellet size as the parameter illustrating the effects of the MPEC.

In the current study, the microscopic‐level comparison between the microparticle‐enriched and standard cultures was conducted for four fungal species, selected on the basis of their growth characteristics, namely *Mucor racemosus*,* Chaetomium globosum*,* Penicillium rubens*, and *A. terreus*. *Mucor racemosus* is a dimorphic fungus representing the group of zygomycetes. Depending on culture conditions, it proliferates as budding yeast‐like cells or branching hyphae and even if it forms pellets, the pellet formation mechanism is nonagglomerative (Orlowski, 1991). *Chaetomium globosum* propagation relies on the formation of asci in dark, hairy perithecia of characteristic appearance (Wang et al., 2016). Actually it is difficult to attribute any mechanism of pellet formation to this species, although spore agglomeration seems to be the most probable. *Penicillium rubens* and *A. terreus* exhibit distinct mechanisms of pellet formation, namely the agglomeration of spores and mycelia, respectively (Grimm et al., 2004; Nielsen, 1996).

The efforts presented in this work were based on the size and shape parameters of mycelial objects computed within the framework of digital image analysis. The aim of the study was to provide a quantitative description of the influence of microparticles on fungi displaying different mechanisms of mycelial development in the submerged conditions.

## 2 MATERIALS AND METHODS

### 2.1 Strains

Four fungal strains obtained from the American Type Culture Collection (ATCC) were tested in the study: *A. terreus* ATCC 20542, *P. rubens* ATCC 9178, *C. globosum* ATCC 6205, and *M. racemosus* ATCC 7924.

### 2.2 Sporulation media

The strains were maintained on slants prepared according to the recommendations of ATCC. For *A. terreus*, the slant medium contained malt extract (20 g·L^−1^) and casein peptone (5 g·L^−1^). *Penicillium rubens* spores were grown on the commercially available potato dextrose medium (BTL Ltd., Poland) containing potato extract (4 g·L^−1^) and glucose (20 g·L^−1^). For *M. racemosus*, the potato dextrose medium was prepared according to the following procedure: 300 g of potatoes was cooked in 500 ml water; potatoes were then discarded and the broth was filled up to 1 L with water; next, glucose (20 g·L^−1^) was added. The slant medium for *C. globosum* was prepared as follows: the commercially available rabbit food pellets (25 g) were boiled in 1 L of water and the filtrate was collected after 30 min of steeping. All the above‐mentioned media were solidified with agar. Spores suspended in sterile water were transferred onto new slants. Then, they were cultivated in the thermostated chamber at 26°C. After 10 days, they additionally remained for 3 days at ambient temperature and were later stored at 4°C. The slants for all studied fungal species were renewed every 2 weeks and the slants of the same age were used in each experiment.

### 2.3 Liquid cultivation media

The tested strains were cultivated in the following liquid media. For *A. terreus* ATCC 20542 and *C. globosum* ATCC 6205, the medium contained lactose (10 g·L^−1^), yeast extract (8 g·L^−1^), KH_2_PO_4_ (1.51 g·L^−1^), MgSO_4_·7H_2_O (0.51 g·L^−1^), NaCl (0.4 g·L^−1^), ZnSO_4_·7H_2_O (1 mg·L^−1^), Fe(NO_3_)_3_·9H_2_O: 2 mg·L^−1^, and biotin 0.04 mg·L^−1^. Additionally, 1 ml·L^−1^ of the trace elements solution containing Na_2_B_4_O_7_·10H_2_O (100 mg·L^−1^), MnSO_4_ (50 mg·L^−1^), CuSO_4_·5H_2_O (250 mg·L^−1^), and Na_2_MoO_4_·2H_2_O (50 mg·L^−1^) was added (Bizukojc & Ledakowicz, 2007; Casas Lopez et al., 2003). The use of lactose as a carbon source for *C. globosum* was previously verified by Prokhorov and Linnik (2011). For *P. rubens* ATCC 9178 (originally deposited in ATCC as *Penicillium notatum*), this medium was modified with regard to the carbon sources and contained lactose (7.5 g l^−1^) and glucose (7.5 g·L^−1^), as glucose is a preferable substrate in the early stages of *P. rubens* growth (Koffler, Emerson, Perlman, & Burris, 1945). *Mucor racemosus* ATCC 7924 was cultivated in preprepared Sabouraud medium (BTL Ltd, Poland) containing peptone (5 g·L^−1^), casein peptone (5 g·L^−1^), and glucose (20 g·L^−1^) (Faramarzi, Badiee, Tabatabaei, Amini, & Torshabi, 2008). All media were sterilized at 121°C for 30 min. It must be mentioned that upon the preliminary experiments, whose results are not presented here, it occurred impossible to apply the same medium composition for all tested strains due to the observed growth limitations.

For the MPEC experiments, 6 g·L^−1^ sterile (separately autoclaved at 121°C for 30 min as dry powder) microparticles of aluminum oxide (Al_2_O_3_) of mean diameter equal to 10 μm were added to the cultivation media. Upon the previous experiments, this amount proved to be high enough to act on filamentous fungi evolution in the submerged culture (Gonciarz & Bizukojć, 2014). At the same time, it was crucial not to add too many microparticles that would aggravate image processing and analysis.

### 2.4 Cultivation conditions

The shake flask culture of 150 ml working volume run in flat‐bottomed flasks was used in each experiment. Fungi were cultivated in the thermostated rotary shaker Certomat^®^ BS‐1 at 28°C (B. Braun Biotech International, contemporarily Sartorius Stedim, Germany). The shaking speed was equal to 110 min^−1^. The cultivations were started as follows. From each slant, the spores of the individual fungus were washed to the appropriate type (medium composition), and amount (dependent on the number of flasks to be prepared) of liquid medium and the spores were enumerated under a microscope (objective 40×) with the use of a Thoma chamber. In each experiment, the initial number of spores in the cultivation medium was maintained at the level of 10^9^ L^−1^. If required, the medium was diluted or more spores were added. For the MPEC runs, the appropriate amount of microparticles was added. Next, the prepared suspension was dispensed to shake flasks. The individual experimental run lasted for 24 hr. Samples were taken every 1, 2, or 3 hr depending on the phase of growth from 5 hr until the end of the experiment. Each MPEC run was accompanied by the standard cultivation, which served as the reference. For each fungus, at least three independent experiments were performed.

### 2.5 Image processing and analysis

The samples from each experiment (standard and MPEC runs conducted in parallel) were subjected to the immediate (no sample storage was applied) microscopic observations. The light microscope OLYMPUS BX53 was used for this purpose (Olympus Corporation, Japan). The microscope was equipped with the high‐ resolution RGB digital camera OLYMPUS DP27 and controlled by the computer with the image analysis software OLYMPUS cellSens Dimension Desktop 1.16 (Olympus Corporation, Japan). Due to fact that the size of the observed mycelial objects changed by several magnitudes, the variety of objectives: 1.25×, 2.5×, 4×, 10×, 20×, 40×, and 100× had to be used. The viable slides were prepared by dropping approximately 10 μl of the fungal suspension and observed using phase contrast. At least 40 RGB images of resolution 2,448 × 1,920 were snapped to assure the minimum number of mycelial objects for analysis (Figure S1).

These images were next subjected to the semiautomatic (actions of the operator incidentally required) image processing and analysis procedures, which were programmed in the macrolanguage of the used software. Their steps were as follows:

1. Filtration of the image with the use of a median filter to smooth the edges of the objects. The median filter does not change either the size or the shape of the objects.
2. Edge detection with the use of a Sobel filter.
3. Image segmentation based on the enhanced edges of the objects to detect the valid mycelial objects in the green plane of the RGB image. The use of the edge detector made the processing and analysis of all images independent of the colors and shades, which were present in the images because of rapidly changing mycelial objects due to their fast growth and consequent changes of objectives (from 40× to 1.25×) to assure the valid magnification.
4. Calculation of the morphological parameters (both size and shape) of the detected mycelial objects:
  ☐ projected area (A) calculated as the pixel count in the given object multiplied by squared calibration unit.
  ☐ mean diameter (D) being the distance of two boundary points on a line through the centre of gravity.
  ☐ elongation (E) defined as the squared quotient of longitudinal and transversal deviation of all pixels belonging to the object along the regression line. If *E* = 1, then the object is ideally circular. The higher the elongation, the more different is the shape of the object from the circle; ultimately, it may become a thin line.
  ☐ roughness (*R*), sometimes called solidity or convexity, calculated as the ratio of projected area to convex area. If *R* = 1, then the object is ideally smooth and convex without any protrusions on its boundary. The values of roughness decrease, when there are any irregularities (like growing out filaments) on its boundary and the object becomes hairy.
  ☐ Morphology number (Wucherpfennig, Hestler, & Krull, 2011) calculated from:
    (1) $$\text{Mo}=\frac{2\cdot \sqrt{A}\cdot S}{\sqrt{\pi }\cdot D_{\max}\cdot E}$$
    where: *A* – mean projected area, *S* – solidity (roughness), *E* – elongation, and *D*_max_ – maximum object diameter. The value of morphology number close to 1 and at the same time not lower than 0.6 is found for the circular objects like ungerminated spores and ideal pellets (pelleted morphology with few clumps and filaments). If the pellets are irregular or filamentous morphology is evolved, the morphology number is lower than 0.5 (Wucherpfennig et al., 2011).
5. Removal of any debris and invalid objects with the use of various size and shape filters: in the images with spores, the removal of noncircular objects upon the value of circularity and in the ones with large pellets the removal too small objects (upon projected area) were required. Some debris had to be manually removed by the operator in this step too. In some images, the areas of interest (AOIs) were manually established to improve the detection of valid mycelial objects and avoid debris originating mainly from microparticles.
6. Transfer of image analysis data into a spreadsheet file.

Additionally, such parameters as the diameter of pellet core or the ratio of core diameter to pellet diameter were measured manually in the selected images.

The parameters of the analyzed mycelial objects were subjected to statistical analysis. The mean values, standard deviation, and confidence band upon *t*‐Student distribution (α = 0.05) were determined. Specifically, in some cases upon mean projected area, two classes of the mycelial objects (smaller and larger ones, regarding their mean projected area) were distinguished by introducing a threshold value (Figure S2). Details are provided in Section 3.

## 3 RESULTS

The quantitative description of spore‐to‐mycelium evolution of four fungal species was presented. The analysis was performed on the basis of microscopic images, examples of which are shown in Figures S3–S6 for the standard cultivation and in Figures S7–S10 for the MPEC.

### 3.1 Growth of *Aspergillus terreus* in the standard and microparticle‐enhanced cultivations

*Aspergillus terreus* conidiospores are spherical and their morphological parameters measured at the start of the standard and MPEC (0 hr) were as follows. The mean projected area took the value of 3 μm^2^ (with the corresponding mean diameter at the level of 2 μm). The elongation and roughness were equal to 1.06 and 0.93, respectively, and the morphology number reached 0.90 (Figure S3a and S7a).

**Figure 1:**
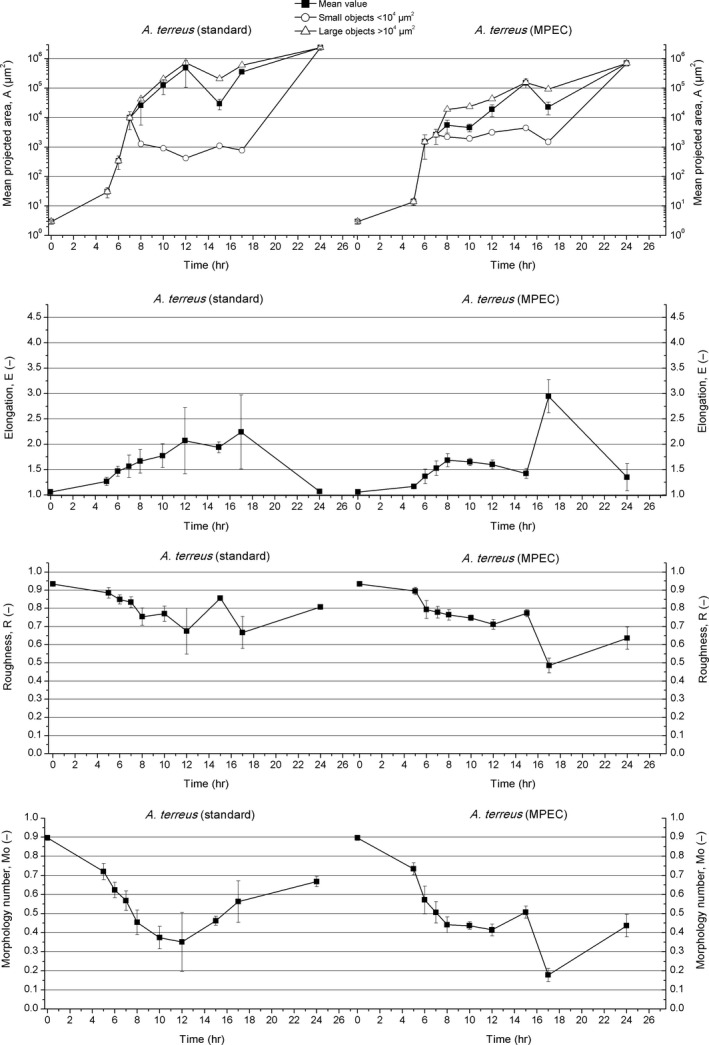
Development of *Aspergillus terreus* in the standard cultivation (left panels) and MPEC (right panels) quantified by morphological parameters

In the first phase of *A. terreus* growth, the swelling of spores took place and looked actually the same irrespective of the type of cultivation. This phase lasted approximately 5 hr and then the mean projected area of individual spores increased to 7 μm^2^ (Figures S3b and S7b). After 5 hr of cultivation, the ongoing process of spores agglomeration was observed. Specifically, the agglomerates composed of several spores were recorded at that time (Figures S3c and S7c). The mean projected area of mycelial objects reached 30 μm^2^ and the morphology number went down to 0.72. These first agglomerates were not ideally circular but rather irregular with not quite smooth edges. The change in shape of these objects led to the increase in elongation to 1.27 with the simultaneous decrease in roughness to 0.88 (Figure 1). Nevertheless, further swelling of spores was noted in concert with spore agglomeration (Figure S3d). Until this moment, the morphological evolution of *A. terreus* in the standard and with MPECs was almost identical.

Later on, in 8 hr of the standard cultivation, the emergence of agglomerates consisting of approximately 4,000 spores (corresponding to *A* = 2.6 × 10^4^ μm^2^) was recorded (Figure S3e). The value of morphology number (equal to 0.45) was the indication of fairly irregular, noncircular shape of agglomerates. Then, two types of objects could have been clearly distinguished out of the mycelial agglomerates (Figures S2 and S3e), namely small agglomerates of mean projected area lower than 10^4^ μm^2^ and large agglomerates of the area exceeding 10^4^ μm^2^. After 8 hr of cultivation, the mean projected area of small agglomerates and large agglomerates reached 1.3 × 10^3^ μm^2^ and 4.2 × 10^4^ μm^2^, respectively (Figure 1).

First germ tubes emerged from the external regions (edges) of the agglomerates not earlier than at 10 hr of the standard cultivation, initiating the next stage of fungal growth, that is, the germination of spores (Figure S3f). The emergence of germ tubes from the agglomerated spores clearly confirmed the agglomerative (aggregative) mechanism of pellet formation in *A. terreus*. The morphology number dropped to 0.37, the elongation was equal to 1.78, and roughness decreased to 0.77 (Figure 1), so the shape of mycelial objects differed from the ideal circle (Figure S3f).

Within the next hours of *A. terreus* mycelial evolution in the standard cultivation, the germ tubes got longer and their transformation into hyphae took place. This was reflected by the decrease in morphology number to the lowest observed level of 0.35 in 12 hr of cultivation (Figure 1). Evolved hyphae covered the cores of the agglomerates and radially grew toward the cultivation medium (Figure S3g). Then, all the agglomerates achieved more regular (more circular) shapes. Almost all objects began to resemble circular or ellipsoid pellets after 15 hr of cultivation (Figure S3h). Due to the change in shape, the elongation decreased to 1.94. At this time, the morphology number increased to 0.46. The mean projected area of small agglomerates and large agglomerates was equal to 1.1 × 10^3^ μm^2^ and 2.1 × 10^5^ μm^2^, respectively (Figure 1).

In 17 hr of the growth in the standard cultivation, *A. terreus* ultimately formed regular hairy pellets with cores of circular or ellipsoid shape and the morphology number of 0.56. Roughness had then its smallest value equal to 0.67, which showed that many filaments grew out of the agglomerates forming a hairy‐like structure around the core of the pellets (Figure S3i). Ultimately, hairy pellets with small cores of regular round shapes and similar size were achieved at the end of the standard cultivation after 24 hr of growth (Figure S3j). The elongation took the value of 1.07 (close to a number corresponding to a circle) and thus returned to the level recorded at the beginning of the experiment. Roughness increased to 0.81 (still quite hairy objects were observed) and the morphology number reached 0.67. The mean projected area was equal to 2.4 × 10^6^ μm^2^ and the two classes of objects of different sizes were not observed any more (Figure 1).

In the case of *A. terreus* growth in the MPEC, many differences were seen from 8 hr of the run compared to the standard cultivation, which is proved by the microscopic images (Figure S7a–j) and the values of morphological parameters (Figure 1). Similarly as in the standard cultivation, two classes of mycelial objects: the ones larger than 10^4^ μm^2^ (large objects) and the ones smaller than 10^4^ μm^2^ (small objects) were also distinguished after 8 hr in the MPEC (Figures S2 and S7e). Nevertheless, the values of mean projected area of the objects belonging to these two classes in the MPEC and in the standard cultivation were different (Figure 1). From 8 hr of the MPEC, the size of objects belonging to small agglomerates took the value between 1 × 10^3^ and 5 × 10^3^ μm^2^ and was much larger than in the standard cultivation (rarely exceeding 1 × 10^3^ μm^2^). On the other hand, the mean projected area of large objects in the MPEC was not higher than 1.6 × 10^5^ μm^2^, while in the standard cultivation it exceeded 7 × 10^5^ μm^2^ (compare Figures S3 f and S7f).

In 10 hr of the MPEC, the mean projected area (calculated for all objects) reached 4.6 × 10^3^ μm^2^, and this value was significantly lower (almost by two magnitudes) than the mean projected area in the standard cultivation, which was equal to 1.3 × 10^5^ μm^2^ (Figure 1). Between 8 and 12 hr, the objects in the MPEC were more circular in comparison with those in the standard cultivation (Figure S7f). This was indicated by the values of elongation (much lower than 2.00) and morphology number (higher than 0.40) in the MPEC (Figure 1), as in the standard cultivation elongation reached 2.07 and the morphology number was remarkably lower (0.35–0.37). Roughness had almost the same values for both A. *terreus* cultivations (Figure 1).

As mentioned above, between 12 and 15 hr of the standard cultivation of *A*. *terreus*, the formation of circular objects (Figure S3g and h) started, since the morphology number increased to 0.46 and the elongation slightly decreased to 1.94 (Figure 1). In the case of the MPEC, where the elongation took the value of 1.42 and the morphology number increased to 0.50, this effect was even more noticeable (Figure S7g and h). Then, between 15 and 17 hr, the values of these parameters fluctuated (Figure 1) and so did roughness, for which the trend of changes was difficult to describe. Images of the objects from these time points are presented in Figure S7h and i.

Finally, after 24 hr of *A. terreus* MPEC cultivation, the pellets with more distinct but smaller cores than those in the standard cultivation were observed (Figure S7j). The mean diameter of the cores in the MPEC was only 218 μm, while in the standard cultivation it reached 418 μm. The ratio of core diameter to mean pellet diameter was also determined. This value was almost identical for the MPEC and for the standard cultivation (0.16 and 0.17, respectively). It clearly corresponds with the structural changes of pellets occurring in the MPEC, where the pellets were far more hairy and with longer filaments growing out of the pellet core. The ratio of filaments to diameter was 0.33, while in the standard cultivation it was only 0.21. Most microparticles were embedded in the structure of the pellets (Figure S7j); however, the core‐shell pellets were not observed. In contrast, the pellets having from 1 to 4 cores (multicore pellets) were observed in the MPEC (Figure S7j). It was another important difference with respect to the standard cultivation. This may indicate that, due to the presence of microparticles, the evenly evolved pellets (the ones with the core formed) had the tendency to loosely agglomerate with each other. This agglomeration was probably taking place with the contribution of long filaments protruding from the pellets. In sum, the mean projected area of MPEC pellets was smaller (even in the case of multicore pellets) in comparison with the standard cultivation. In 24 hr of the run, the elongation parameter, after certain fluctuations, reached 1.35. This value was higher than the one recorded in the standard cultivation, what demonstrates that MPEC pellets were also less circular (Figure S7j). The morphology number was equal to 0.44 and was lower than in the standard cultivation. The addition of Al_2_O_3_ microparticles in the MPEC also changed the structure of *A. terreus* pellets, which was reflected by the value of roughness (0.64). Pellets in the standard cultivation were smoother, as their roughness was at the level of 0.81 (Figure 1). In addition, few dispersed mycelia, namely branched and unbranched hyphae and clumps, were visible (Figure S7j).

### 3.2 Growth of *Penicillium rubens* in the standard and microparticle‐enhanced cultivations

**Figure 2:**
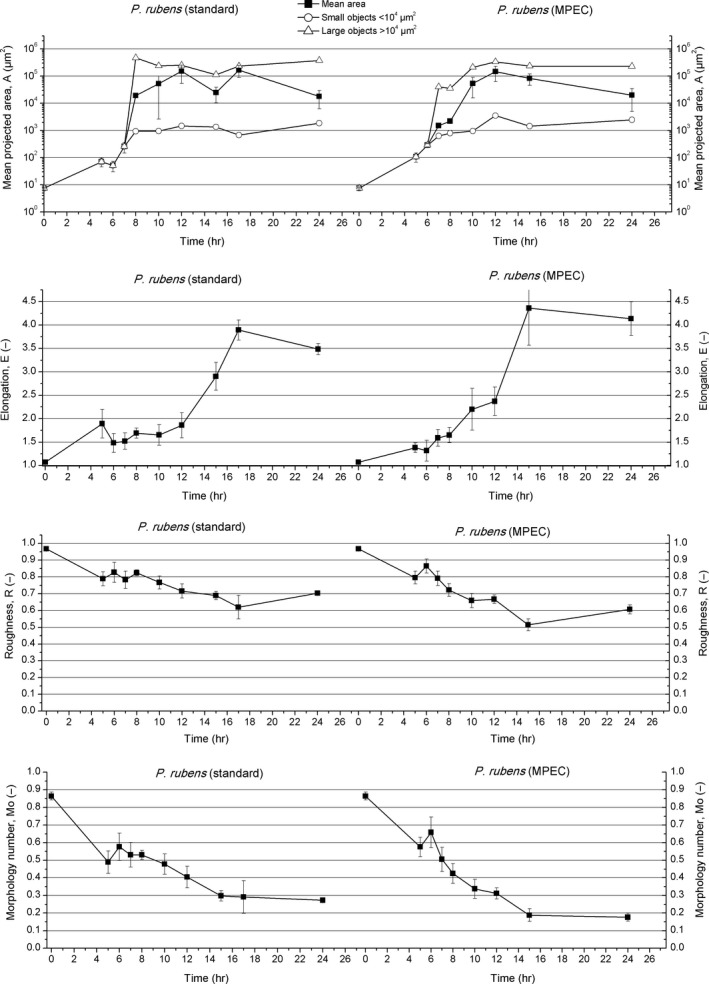
Development of *Penicillium rubens* in the standard cultivation (left panels) and MPEC (right panels) quantified by morphological parameters

In contrast to *A. terreus*, the studied cultures of *P. rubens* were initiated from slightly elongated spores. Their mean projected area was at the level of 8 μm^2^ (with the mean diameter equal to 3 μm). The spores were visible as smooth objects and the corresponding roughness parameter took the value of 0.97. The morphology number and elongation were equal to 0.86 and 1.07, respectively (Figure S4a, Figure 2). The simultaneously occurring events of spore swelling and germination constituted the first stage of *P. rubens* mycelial evolution. Single germ tubes and spores, with the area varying greatly from 8 to 20 μm^2^, were observed after 5 hr of growth (Figure S4b). The morphology number dropped to 0.49 and the elongation increased to 1.89. Roughness went down to 0.79 and its decrease (with slight fluctuations) proceeded continuously during the next 15 hr of the standard cultivation (Figure 2).

In the following hours of the standard cultivation, the agglomeration took place and the first agglomerates composed of several germinated spores could be observed (Figure S4c and d). Afterwards the agglomerates grew, achieving irregular shapes (elongation parameter equal to 1.69) and various sizes (Figure 2). Similarly as for *A. terreus*, the mycelial objects in *P. rubens* culture were grouped into two size classes. The first class comprised the irregular agglomerates (exceeding 10^4^ μm^2^) of spores mainly covered by elongating germ tubes. The second class included the dispersed hyphae of mean projected area lower than 10^4^ μm^2^ (Figure S4e). More specifically, the mean projected area of agglomerates and dispersed hyphae was equal to 4.6 × 10^5^ μm^2^ and 930 μm^2^, respectively. In 10 hr of the standard cultivation, practically all *P. rubens* spores visible under the microscope had their germ tubes emerged (Figure S4f). Within the next hours, hyphae elongation and hyphal branching took place. Branching hyphae covered the larger agglomerates and the class of smaller objects (of the mean projected area lower than 10,000 μm^2^) comprised only individual germ tubes, branched and unbranched hyphae, clump forms and agglomerates composed of less than 10 germinated spores (Figures S4h and S2).

First, hairy pellets of regular, ellipsoid shape were observed after 17 hr of *P*. *rubens* growth in the standard cultivation (Figure S4i). Their mean projected area was equal to 2.3 × 10^5^ μm^2^. However, branched, unbranched hyphae and clumps prevailed, increasing the elongation to 3.89 and decreasing the morphology number to 0.29. The lowest value of roughness, equal to 0.62, was also observed. Since that point, roughness started to increase again (Figure 2).

At the end of the standard cultivation (after 24 hr), hairy pellets of more or less ellipsoid shapes and mean projected area equal to 3.8 × 10^5^ μm^2^ were developed (Figure 2, Figure S4j). Still, dispersed hyphae dominated. Their mean projected area took the value of 1.8 × 10^3 ^μm^2^. The presence of objects characteristic for fungal dispersed morphology resulted in the lowest morphology number of 0.27 and the decrease in elongation to 3.48, whereas the roughness parameter reached 0.70 (Figure 2).

The effect of microparticles on *P. rubens* growth and morphology (Figure S8a–j) was as follows. The agglomeration stage was accelerated by 1 hr with respect to the standard culture (Figure 2) and the first agglomerates composed of several germinated spores appeared in 5 hr of *P. rubens* growth (Figure S8b). This resulted in the difference of mean projected area between the standard cultivation (70 μm^2^) and the MPEC (107 μm^2^). Until the 5 hr of cultivation, the change in elongation in the MPEC was not as significant as in the standard run, with the corresponding parameter values reaching 1.38 in the MPEC and 1.89 in the standard cultivation (Figure 2). Roughness was almost the same in both cultivations, being equal to about 0.80 in both runs. The morphology number was equal to 0.57 and was lower in the MPEC than in the standard cultivation. In 5 hr of the standard cultivation, this parameter was equal to 0.49 (Figure 2).

Similarly as in the standard run, two classes of objects, namely the ones with area exceeding 10^4^ μm^2^ (large objects) and the ones with area lower than 10^4^ μm^2^ (small objects) were distinguished (Figure S2). However, in the MPEC, they were found 1 hr earlier (in 7 hr).

The first regular circular agglomerates were observed in 10 hr of the MPEC culture. Unlike the standard cultivation, in the MPEC, the hyphae covering the agglomerates were shorter (Figure S8f). The mean length of filaments covering the agglomerates in the MPEC was equal to 12 μm, whereas in the standard cultivation it reached 20 μm. Mean projected area of agglomerates for both cultivations was approximately equal to 5 × 10^4^ μm^2^ (exact value for the MPEC was 5.3 × 10^4^ μm^2^). The elongation in the MPEC and the standard cultivation were 2.2 and 1.65 (Figure 2), respectively. Clearly, the elongation for the MPEC was significantly higher. The objects in the standard cultivation were smoother as roughness in the MPEC was lower (0.66). The morphology number in the MPEC was not as high as in the standard cultivation (Figure 2).

The first oval hairy pellets with the distinct cores were observed in 15 hr of the MPEC, 2 hr earlier than in the standard cultivation (Figure S8h). Moreover, at this stage, many branched, unbranched hyphae, and clumps were seen (Figure S8h). Accordingly, the highest elongation equal to 4.35 was noted in 15 hr of the MPEC. Morphology number and roughness reached their lowest values equal to 0.19 and 0.51, respectively (Figure 2). The intensive filamentous hyphae growth was observed until the end of the experiment (Figure S8i).

In 24 hr of the MPEC, besides dispersed hyphae, hairy pellets of nonideal ellipsoid shape were developed (Figure S8i). The morphology number took the value of 0.18. Furthermore, pellets formed in the MPEC were similar in structure and shape to the pellets grown without microparticles. With regard to size, it is impossible to unequivocally state which of them were bigger. Taking mean projected area of objects of both classes into account, the objects from the MPEC were astonishingly bigger (*A* = 1.9 × 10^5^ μm^2^) that the ones from the standard culture (*A* = 1.7 × 10^5^ μm^2^). However, if large objects (pellets) were taken into account (Figure 2), they were smaller in the MPEC (*A* = 3.7 × 10^6^ μm^2^ vs. *A* = 2.3 × 10^6^ μm^2^). Small objects from the standard culture were smaller (*A* = 1.8 × 10^3^ μm^2^) than those from the MPEC (2.5 × 10^3^ μm^2^). So, it seemed that the action of microparticles depended on the size of objects. It led to decreased size of larger objects and increased size of smaller ones. The similar effect was also noticed for *A*. *terreus* (see Section 3.2.1). Microparticles somewhat stabilized the agglomerates in *P*. *rubens*. In 24 hr of the MPEC, the roughness parameter reached 0.61 and was lower than in the standard cultivation (0.7), which meant that the objects were more hairy and probably of more loose structure (Figure S8i).

### 3.3 Growth of *Chaetomium globosum* in the standard and microparticle‐enhanced cultivations

**Figure 3:**
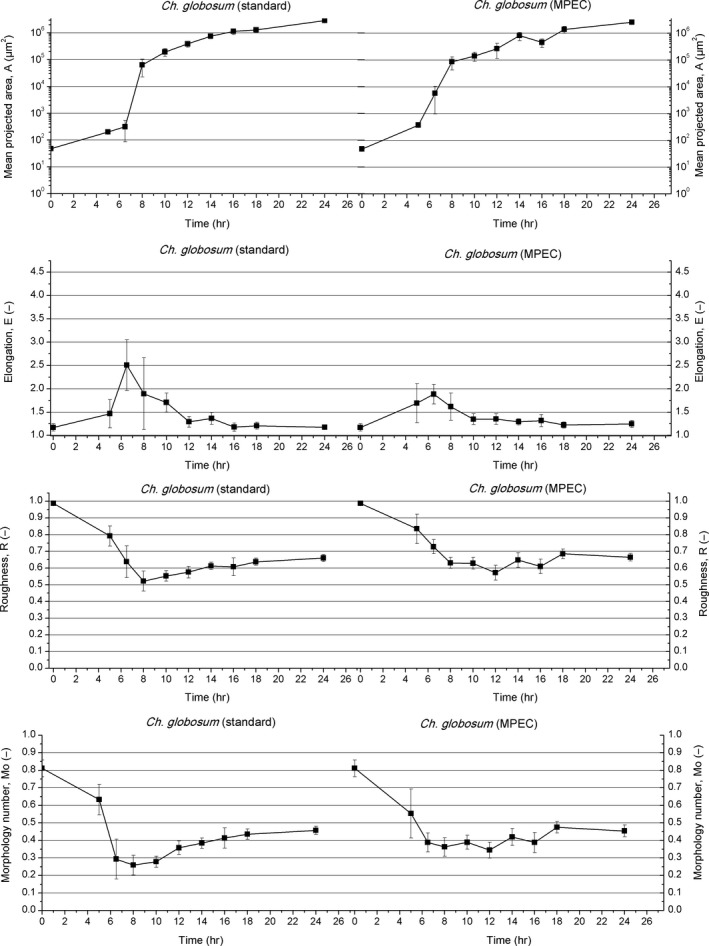
Development of *Chaetomium globosum* in the standard cultivation (left panels) and MPEC (right panels) quantified by morphological parameters

The standard and MPECs of *C. globosum* were started from large, elongated, lemon‐shaped spores (Figure S5a) with the mean projected area of 48 μm^2^ and the mean diameter equal to 8 μm. The morphology number and elongation parameters were equal to 0.81 and 1.17, respectively. Roughness reached almost the same level as for *A. terreus* and took the value of 0.99 (Figure 3).

Within the first 5 hr of both cultivations of *C. globosum*, its spores swelled and germinated (Figures S5b and S9b). In the standard cultivation, their mean projected area was equal to 202 μm^2^. The elongation, morphology number, and roughness reached 1.47, 0.63, and 0.79, respectively (Figure S5b, Figure 3). Subsequently, germinated spores had their germ tubes elongated and filamentous hyphae (branched and unbranched hyphae, clump forms) were formed. A limited number of agglomerates was observed at this point (Figure S5c). In 6.5 hr of *C. globosum* standard cultivation, the highest level of elongation was achieved, with the corresponding parameter value peaking at 2.51.

More agglomerates of mean projected area 6.3 × 10^4^ μm^2^ occurred after 8 hr of the standard cultibvation (Figure S5d). Germinated spores agglomerated mainly around hairy perithecia, which became the agglomeration centers for future pellets. Spores which had not been taken out of the perithecia germinated inside them, tearing up their structure. A decline of elongation was initiated as this parameter dropped to 1.89. Morphology number and roughness reached the lowest levels in *C. globosum* mycelial evolution, decreasing to 0.26 and 0.52, respectively. Within next hours, filamentous hyphae elongated and the agglomeration process took place (Figure S5e). Only one size class of objects was observed both the standard cultivation and in the MPEC (Figure S2). In 12 hr of the standard cultivation of *C. globosum*, the first regular pellets of circular shape were observed (Figure S5f). Their mean projected area reached 3.9 × 10^5^ μm^2^, the elongation was equal to 1.29, and the morphology number raised to 0.36 (Figure 3). Roughness increased steadily up to 0.58. Within the next 4 hr of the cultivation, the mean projected area was successively rising due to hyphal agglomeration and proliferation. The pellets grew and acquired a more and more circular shape, what is seen in Figure S5g–i.

After 24 hr of the standard cultivation, the mean projected area of *C. globosum* pellets increased to 2.9 × 10^6^ μm^2^. The elongation parameter fluctuated and ultimately reached the value of 1.18. The morphology number took the value of 0.46. Roughness was equal to 0.66 (Figure 3), what reflected the irregular (hairy) edges of the pellets (Figure S5j). Finally, hairy pellets with relatively loose, not distinct cores were developed. Hyphae seemed to be uniformly distributed along the pellet. No filamentous hyphae (branched, unbranched hyphae, or clumps) were recorded and only one class of objects (with regard to size) was distinguished in the course of *C. globosum* cultivation. (Figure S2).

The effect of microparticles on *C. globosum* growth and morphology is visualized in Figure S9a–j. The stage of agglomeration of spores was accelerated by 1.5 hr, and the dramatic change in the internal structure of agglomerates and evolved pellets compared to the standard culture was noticed. Nonregular clump‐like agglomerates were observed in 6.5 hr of the MPEC cultivation (Figure S9c). Their mean projected area was equal to 5.7 × 10^3^ μm^2^, while in the standard cultivation it was only 315 μm^2^ (Figure 3). The elongation in 6.5 hr in the MPEC and in the standard cultivation reached the highest value of 1.89 and 2.50, respectively. Afterwards, the elongation started to decrease until the end of both cultivations (Figure 3). Roughness and the morphology number in the MPEC and the standard cultivation were decreasing from the start until 8 hr of the experiment. This decrease was more rapid in the standard cultivation. At this point, roughness in the MPEC cultivation was 0.63 and in the standard cultivation it dropped to 0.52. The morphology number in the MPEC (equal to 0.36) was also higher than in the conventional cultivation (0.26). After 8 hr this trend changed. In the MPEC, fluctuations (with the increasing trend) of roughness and the morphology number occurred. In the case of the standard cultivation these two parameters rose gradually (Figure 3).

The first regular ellipsoid pellets with the distinct cores were formed in 10 hr of the MPEC, that is, 2 hr earlier in comparison with the standard cultivation (Figure S9e). Their mean projected area was equal to 1.4 × 10^5^ μm^2^ and they were smaller than those in the standard cultivation. Pellets formation caused the slight increase in morphology number to 0.39 and decrease in elongation to 1.35 (Figure 3). In light of the fact that in the standard cultivation such pellets were not yet present at this point, the morphology number was lower and the elongation was higher. Roughness did not significantly change from 8 hr of the MPEC and its value was equal to 0.63. Compared with the standard cultivation the MPEC objects were smoother and their roughness was higher. After 12 hr of both *C. globosum* cultures, the elongation parameter remained at the level of about 1.25 until the end of the standard cultivation and MPEC (Figure 3).

Pellets that were ultimately formed in 24 hr of *C. globosum* MPEC were described as the core‐shell pellets covered with long filaments (Figure S9j). The corresponding morphology number was equal to 0.45 (Figure 3). Interestingly, this value was almost identical for the standard cultivation. It indicated that the main effect of microparticles was associated with the formation of the mineral core in the pellets. The cores of the pellets from the standard cultivation were less distinct (Figure S5j). The hyphae forming the pellet were uniformly distributed. In contrast, the pellets of *A. terreus* had visibly denser hyphae in their centers. (Figure S3j). *C. globosum* pellets from the MPEC were almost of the same size (*A* = 2.6 × 10^6^ μm^2^) as in the standard cultivation (*A* = 2.9 × 10^6^ μm^2^). The elongation in the MPEC reached the value of 1.25 and was higher than in the standard cultivation (1.18). Roughness in the MPEC was ultimately equal to 0.67 and this value did not significantly differ from the one determined for the standard culture (Figure 3).

### 3.4 Growth of *Mucor racemosus* in standard and microparticle‐enhanced cultivations

**Figure 4:**
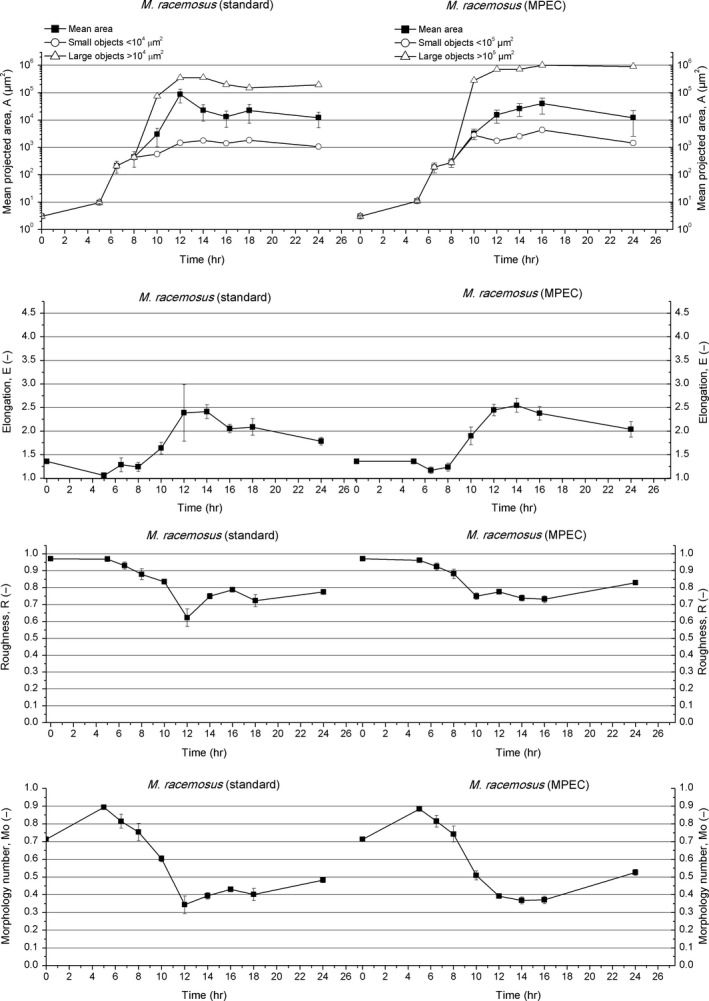
Development of *Mucor racemosus* in the standard cultivation (left panels) and MPEC (right panels) quantified by morphological parameters

The spores of *M. racemosus*, from which the standard and microparticles‐enhanced cultivations started, were ellipsoid (Figures S6a and S10a) and their sizes varied from 2 to 6 μm^2^ (the mean diameter equal to 2 μm). The morphology number, equal to 0.71, was lower than the one observed for *A. terreus* and *P. rubens*. The elongation parameter reached the value 1.36, which was higher compared to *A. terreus* and *P. rubens*. Roughness was equal to 0.97 (Figure 4).

The first stage of *M. racemosus* growth in the standard cultivation comprised the rapid process of spores swelling leading to the change in spore shape into almost ideal circles with the values of morphology number, elongation, and roughness equal to 0.89, 1.06, and 0.97, respectively. Within 6.5 hr of the experiment, the spore size increased by two orders of magnitude exceeding 200 μm^2^ (Figure S6b).

After 6.5 hr of the standard cultivation, the next stage of *M. racemosus* growth began, namely the germination of spores (Figure S6c). The process of germination was accompanied by the decrease in roughness. Notably, some spores remained dormant. The mean projected area of mycelial objects was equal to 210 μm^2^, the morphology number slightly decreased to 0.81, and the elongation reached 1.29 (Figure 4). Within next hours, germ tubes emerged and subsequently transformed into branched hyphae (Figure S6d). No agglomeration was observed at this point.

More complex clump forms occurred after 10 hr of the standard cultivation. Then, two types of objects could be clearly distinguished (Figure S6e)—small mycelial objects (branched and unbranched hyphae) of mean projected area lower than 10^4^ μm^2^ and clumps of area exceeding 10^4^ μm^2^ (Figure S2). The mean projected area was exactly equal to 569 μm^2^ for small objects and 7.5 × 10^4^ μm^2^ for clumps in 10 hr of the run. Ultimately, almost all spores germinated after 12 hr of *M. racemosus* standard cultivation. Two to five germ tubes from each spore were observed (Figure S6f). At this point, the lowest roughness value equal to 0.62 was recorded (Figure 4). Later on, filamentous forms continued to elongate and more numerous agglomerates of mean projected area of 3.5 × 10^5^ μm^2^ appeared. The agglomerates were of rather loose structure (Figure S6g) and were categorized as clumps. The elongation reached 2.39 and the morphology number took the value of 0.34. Overall, *M. racemosus* exhibited a large variety of morphological forms in 14 hr of the standard cultivation, namely the spores with germ tubes, filamentous branched hyphae and clump‐like large agglomerates (Figure S6g). Roughness fluctuated around 0.75.

Some large clumps began to resemble pellets (with a visible core‐like area) after 16 hr of *M. racemosus* standard evolution (Figure S6h and i). Then, the elongation went down to 2.06 and roughness increased to 0.79 (Figure 4). After 24 hr of the standard cultivation, few hairy pellets of irregular shapes were seen. Nevertheless, the loose‐structure clumps (without distinct cores) dominated (Figure S6j), resulting in further decrease in elongation down to 2.04. The mean projected area of agglomerates (they comprised the class of larger objects) reached 2 × 10^5 ^μm^2^. However, dispersed morphological forms like branched hyphae and small clumps prevailed. Their mean projected area did not exceed 1.1·10^3^ μm^2^. Ultimately, morphology number and roughness reached 0.48 and 0.77, respectively (Figure 4).

The mineral microparticles in the MPEC had a noteworthy influence on *M. racemosus* growth and morphology (Figure S10a–j). Above all, they forced *M. racemosus* toward the formation of pellets with distinct cores (Figure S10i). It was the most significant outcome of microparticles addition.

The first stage of *M. racemosus* proliferation, namely spore swelling, was slightly slower in the MPEC in comparison with the standard cultivation. Within first 6.5 hr of the MPEC, the mean projected area of spores reached only 190 μm^2^, while in the standard cultivation it exceeded 200 μm^2^ (Figure 4). In the MPEC, the elongation reached its minimal value equal to 1.17, which corresponded to the most spherical shape observed for *M. racemosus* objects. The elongation in the MPEC was also lower than in the standard cultivation (the minimum was achieved earlier in 5 hr). At this point, the spores in the standard culture already began to elongate (Figure S6b).

The formation of *M. racemosus* agglomerates in the MPEC started in 10 hr of growth. Two classes of objects were distinguished (Figure S2). The sizes of these classes were completely different from the ones observed for the standard culture. It was the only case out of all studied cultivations that the threshold value was at the level of 10^5^ μm^2^, not 10^4^ μm^2^ (Figure S2). The mean projected area for the large agglomerates was equal to 7.5 × 10^4^ μm^2^ in the standard cultivation and 2.9 × 10^5^ μm^2^ in the MPEC (Figure 4). The increase in the mean projected area of objects was the main effect of microparticles addition to *M. racemosus* cultures. The values of morphology number and elongation parameter reflected the fact that in 10 hr of the MPEC all objects were more elongated than those in the standard cultivation. The morphology number in the MPEC was lower (Mo = 0.51 in the MPEC and Mo = 0.60 in the standard cultivation) and the elongation reached a higher value (*E* = 1.90 in the MPEC and *E* = 1.64 in the standard cultivation). In addition, smoother objects were recorded in the standard cultivation, since in the MPEC a higher value of roughness parameter was obtained (*R* = 0.75). All these differences are presented in Figure 4.

Objects resembling pellets were formed in 12 hr of *M. racemosus* growth. This event took place 4 hr earlier than in the standard cultivation (Figure S10f). The mean projected area of the large objects class in the MPEC continued to increase (7.1 × 10^5^ μm^2^) and was still higher than in the standard cultivation. The elongation and morphology number were similar in both cultivations. In the MPEC, their exact values were equal to 2.45 and 0.39, respectively. Roughness in the MPEC reached 0.78 and was higher than in the standard cultivation (Figure 4).

In 16 hr of the MPEC, the morphology number was equal to 0.37. At the same time in the standard cultivation, its value was equal to 0.43. The mean projected area of the objects calculated for both classes continued to increase (*A* = 4 × 10^4^ μm^2^). Roughness slightly decreased to 0.73 and was lower than that in the standard cultivation (Figure 4). The elongation in the MPEC reached 2.38. Notably, the objects in the standard cultivation were less elongated (*E* = 2.06) (Figure 4).

Numerous pellets with distinct cores, which were observed in 24 hr of *M. racemosus* MPEC, had rather irregular shapes (Figure S10i). This was reflected by the value of morphology number equal to 0.53. It is worth mentioning that the morphology number in the standard cultivation was much lower than 0.5, indicating the presence of dispersed mycelium rather than the pelleted morphology. The elongation in the MPEC of *M. racemosus* reached 2.04 and was higher than these values observed for *A. terreus*,* C. globosum* or *P. rubens*. The mean projected area of large agglomerates in the MPEC cultivation was equal to 9 × 10^5^ μm^2^, whereas in the standard cultivation this parameter had a lower value of 2 × 10^5^ μm^2^ (Figure 4). Roughness in the MPEC cultivation was also higher than in the standard cultivation and reached 0.83. Again, the observed effect of microparticles was far from typical in this case, as one would normally expect the size of agglomerates and pellets to be lower in the MPEC than in the standard submerged cultures.

## 4 DISCUSSION

All the experiments carried out in this study focused only on the mycelial evolution, and interactions of microparticles and mycelia of various fungal species. As the most important events of fungal evolution took place during the first 24 hr of growth in the preculture, fungal products, either secondary metabolites or enzymes, were not detectable in the broth in the end of each experiment. It is the reason why product concentrations were not determined in this study, despite the well‐known fact that the MPEC is used to increase fungal productivity.

With regard to the behavior of the tested fungal species in the studied submerged cultures, only the results obtained for *A. terreus* were clearly in accordance with the mechanism of pellet formation by spore agglomeration described in literature (Bizukojc & Ledakowicz, 2010; Metz & Kossen, 1977). Two stages of pellets formation were distinguished. In the first stage, two size classes of agglomerates (area above and under 10^4^ μm^2^) were observed. In the second stage, all objects agglomerated, creating round shaped, hairy pellets. A similar two‐stage agglomeration process was previously recorded for *A. niger*. In the first stage of the corresponding study, 10% of spores present in the cultivation medium agglomerated. Afterwards, the spores germinated and entered the second stage of agglomeration. As a result, over 90% of the *A. niger* spores agglomerated into pellets (Grimm et al., 2004).

*Penicillium rubens* displayed the pure hyphal agglomeration mechanism (Duckworth & Harris, 1949; Metz & Kossen, 1977). The agglomeration started after germination of spores and resulted in hardly spherical pellets, much different from those formed by *C. globosum* or *A. terreus* (Nielsen, Johansen, Jacobsen, Krabben, & Villadsen, 1995). Two classes of objects were distinguished. However, unlike for *A. terreus*, these two classes were observed in the broth until the end of the 24‐hr cultivation period. Ultimately, the dispersed hyphae dominated over the pellets. This type of morphology was somewhat a mixture of large pellets and small hyphal elements.

Although *C. globosum* formed fairly elegant circular hairy pellets in the end of the experiment, its morphological evolution more resembled the hyphal agglomeration scheme typical for penicilli. Nongerminated spores hardly agglomerated with each other but had the affinity to the remnants of hairy perithecia. In fact, they often germinated before agglomeration. Some of them germinated inside the perithecia. Furthermore, their germ tubes emerged much earlier that the ones in *A. terreus*. The germinated spores with elongating hyphae formed the hairy pellets. Their shape was similar to the one observed for *A. terreus*, but completely different from the shape of *P. rubens* pellets. It is difficult to discuss these observations with literature as no references concerning the early stages of *C. globosum* growth have been published so far.

Last but not least, a nonagglomerative lower fungus *M. racemosus* ultimately formed a limited number of pellets; however, they were much smaller and, above all, looser than those of *A. terreus*,* C. globosum* and *P. rubens*. These pellets were also formed from a much lower number of germinated spores (hyphal elements) than those of the spore‐ or hyphae‐agglomerative fungi. The mechanism of *M. racemosus* pellet formation was similar to the one described for an actinobacterium *Streptomyces tendae*. According to Vecht‐Lifshitz, Magdassi, and Braun (1990), *S*. *tendae* exhibited a nonagglomerative mechanism of pellets formation. The agglomeration of mycelium occurred if the number of spores in the inoculum exceeded 10^6^ L^−1^. As a result, pellets containing not more than 300 germinated spores were formed and this number is at least by two magnitudes lower than for agglomerative fungal species.

In previous literature reports, the effect of mineral microparticles used in the MPEC has actually been attributed to the decrease in the size of agglomerates (pellets) and change in their structure toward the looser one (Krull et al., 2013). However, the majority of experiments involved the spore‐agglomerating species (mostly *Aspergillus*) and any discussions or data regarding other species are hardly found (Kaup et al., 2008).

The experiments performed here revealed the diversity of morphological scenarios observed among filamentous fungi in the MPEC. Importantly, the generalized thesis regarding the decrease in agglomerates size was shown to be only partially true. It could have been somewhat expected, since the interspecies differences in the early evolution of mycelia were already found in the standard submerges cultures. In sum, in the case of spore‐agglomerating *A. terreus*, the effect of microparticles contributed to the decrease in pellets size, as previously observed in *Aspergilli*, including *A. terreus*, by Driouch et al. (2012), Gonciarz and Bizukojć (2014), Coban et al. (2015a, 2015b), Etschmann et al. (2015), Gonciarz, Kowalska, and Bizukojć (2016). Even though the dispersed morphology was previously reported for *Aspergillus* (Driouch, Sommer, et al. 2010), it was not observed in the present study. Nevertheless, the change in structure with respect to the non‐MPEC cultures was apparent and involved the growth of longer and looser filaments. The effect on *C. globosum* was similar; however, in this case, the formation of core‐shell pellets was recorded. Similar pellets were previously described for *A. niger* cultivated in the presence of titanate microparticles (Driouch et al., 2012).

Aside from the “destructive” action of mineral microparticles toward *A. terreus* and *C. globosum* agglomerates, the opposite, yet unknown, effects were also observed. *P. rubens* formed more stable pellets as the hyphal agglomeration in this species was somewhat strengthened. It is a new finding, since the detailed research on the MPEC of penicilli have not been conducted so far.

Aluminum oxide microparticles promoted the formation of pellets with distinct cores in the nonagglomerative *M. racemosus*. Until now, only two MPEC‐related literature reports on zygomycetes were published. Specifically, these studies aimed at characterizing the growth of *Mortierella isabelina* and *Rhizopus oryzae* (Gao et al., 2014; Coban and Demirci, 2016). For *Mortierella isabellina*, unlike *M. racemosus*, the formation of small pellets and the decrease in their size due to the presence of microparticles was observed (Gao et al., 2014). The case of *Rhizopus oryzae* was more interesting and complicated. This nonagglomerating fungus grew as bulk, dispersed mycelium, but in the certain range (around 10 g·L^−1^) of talc or aluminum oxide microparticles the formation of pellets took place (similarly as for *M. racemosus* tested here). Interestingly, with the further increase in microparticles concentration the culture returned to a more dispersed morphology (Coban and Demirci, 2016). Nevertheless, in light of previous literature findings, the stabilizing effect of microparticles on pellets is plausible. For example, this mechanism was previously observed for agglomerative *A. terreus* growing under high shear stress in the bioreactor culture (Gonciarz et al., 2016).

In sum, when applying microparticles one must be aware that their action toward filamentous fungi is species‐dependent. It is also crucial to understand the consequences of morphological changes with regard to aeration, mixing and mass transfer conditions and resulting increase (or undesired decrease) of the productivity of the culture. The tests presented here were performed solely for precultures and it was clearly demonstrated that microparticles contributed to the development of a particular morphological form. No matter whether the agglomerates are formed or not or if their size increases or decreases, the application of microparticles is fully justified as long as the resulting morphological form is favorable in terms of bioprocess performance. The correlation between morphological form and the production of metabolites and enzymes needs to be studied individually for each bioproduct and species. This individual approach to the MPEC was also previously emphasized by Etschmann et al. (2015).

Despite the need for the aforementioned individual approach and a lack of products formed in the precultures studied here, one may speculate on the optimum fungal morphology of tested species and plausible positive outcomes of the use of these precultures as the inocula. Generally, the fact that the pellets are smaller and looser makes them more productive with regard to secondary metabolites and enzymes. It is the case of lovastatin producer *A. terreus* (smaller and less dense pellets) and cellulolytic enzymes producer *C. globosum* (smaller core‐shell pellets with thin mycelial layer around the core). Diffusion of oxygen in such pellets is more effective, as it was many times shown in literature (increase in lovastatin production, Gonciarz et al., 2016), (core‐shell pellets favoring β‐fructofuranosidase production, Driouch et al., 2012). The formation of secondary metabolites and hydrolytic enzymes is catabolism‐dependent and requires significant supplies of energy and thus oxygen. With regard to the penicillin‐producer *P. rubens*, the structure of pellets was stabilized, although the size of the pellets was slightly altered. More stable pellets might decrease the viscosity of broths and increase the convective oxygen transfer. Formation of micropellets (not macropellets) of little diffusion resistance by *M. racemosus* may be favorable for the overall run of the growth process, as the convective oxygen transfer for highly dispersed and, at the same time, highly viscous fungal suspensions is weak. Less viscous micropellets suspension will increase convective oxygen transfer in the bioreactor. *Mucor racemosus* is not a profound producer of metabolites (it was tested here as the representative of lower fungi); however, it does biotransform steroids in the oxygen‐dependent reactions (Faramarzi et al., 2008) and the changes in morphology observed in the present work might be advantageous in this context.

Ultimately, the findings from the presented experiments may prove valuable for those who work with the same fungal genera, leaving alone the fact that the precultures thoroughly studied here are going to be used in our future experiments as the inocula for the larger scale bioreactor processes.

## 5 CONCLUSIONS

Upon the performed experiments, three general conclusions can be drawn:

- The action of microparticles in the MPEC seems to be dependent on the size of mycelial objects.
- Microparticles may accelerate and favor agglomeration of mycelium, as the spores and small mycelial objects (like free‐branched and unbranched hyphae) have the affinity to microparticles, what ultimately results in a kind of scaffold for the construction of the agglomerates.
- Unlike in the case of small mycelial objects, microparticles make the agglomeration of the larger mycelial objects more difficult, sometimes destroying the agglomerates, what leads to the decrease in pellet size.

Regarding the individual species one can conclude that:

- As *A. terreus* is a typical spore‐agglomerative fungal species, microparticles addition caused the decrease in pellets size and changed the internal pellet structure. It also contributed to prolonging the agglomeration of small pellets, resulting in the formation of multicore pellets.
- In the case of *P. rubens*, a typical hyphal agglomerative species, microparticles addition accelerated the agglomeration of hyphae but hardly influenced the size of the evolved pellets.
- In spite of the fact that the mechanism of pellet formation in *C. globosum* cannot be categorized as spore‐agglomerative or hyphal agglomerative, the effect of microparticles was in this case similar to the one observed for spore‐agglomerative *A. terreus*. Microparticles addition accelerated the agglomeration stage, decreased the size of the pellets, and also led to the development of core‐shell pellets.
- The significant effect of microparticles on *M. racemosus* was observed. This nonagglomerative species formed the noticeable number of pellets with distinct cores due to the presence of microparticles.

## CONFLICT OF INTEREST

None declared.
