# Supplementary material for: Morphological evolution of various fungal species in the presence and absence of aluminum oxide microparticles: Comparative and quantitative insights into microparticle‐enhanced cultivation (MPEC)
Source: Microbiologyopen. 2018 Mar 5;7(5):e00603. doi: 10.1002/mbo3.603 (PMC6182563; doi:10.1002/mbo3.603)
Supplement: Supplementary file 1 [file MBO3-7-e00603-s001.docx]

**Supplementary Material**

**MicrobiologyOpen**

**Morphological evolution of various fungal species in the presence and absence of mineral microparticles: comparative and quantitative insights into microparticle-enhanced cultivation (MPEC)**

Anna Kowalska*^a^, Tomasz Boruta^a^, Marcin Bizukojc^a^

^a^ Lodz University of Technology, Faculty of Process and Environmental Engineering, Department of Bioprocess Engineering, ul. Wolczanska 213, 90-924 Lodz, Poland, phone: +48 42 631 39 77, fax +48 42 636 56 63 e-mail: pani.anna.ks@gmail.com

* Corresponding author

**Supplementary Fig. 1.** Estimation of the number of objects required to calculate the mean value of the morphological parameters (mean projected area used as an example)

**Supplementary Fig. 2.** Distinction, regarding mean projected area, of two classes of objects for the studied filamentous fungi during first 24 hours of their evolution in the submerged cultures (standard and MPEC)


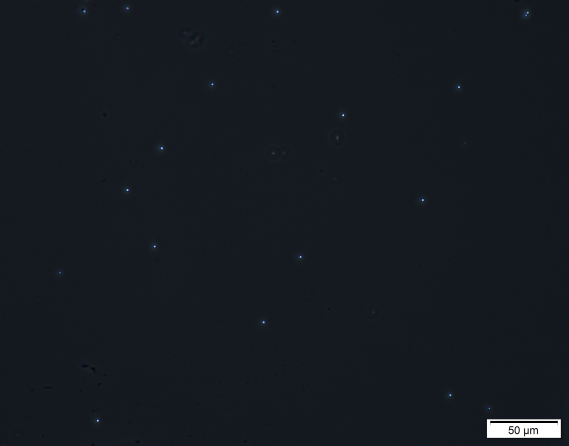

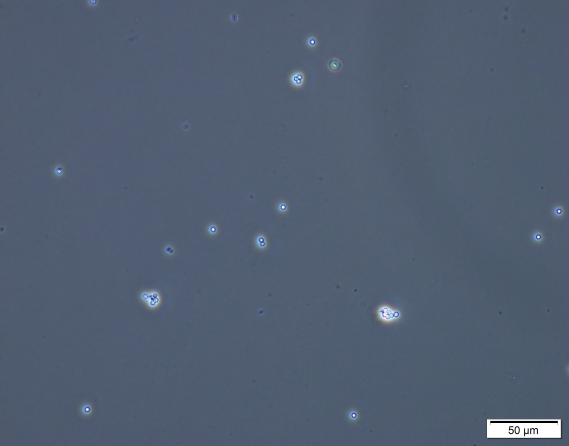

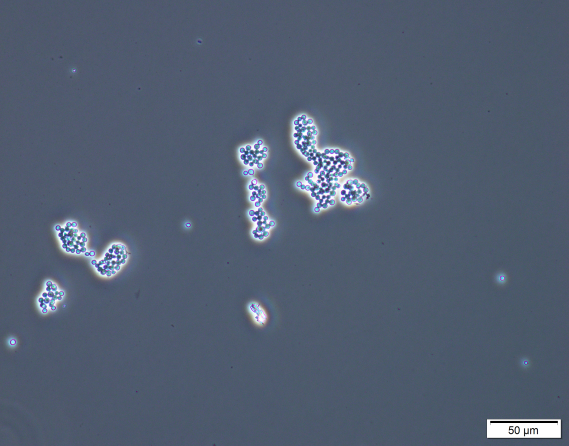


**c**

**b**

**a**

**d**

**f**

**e**


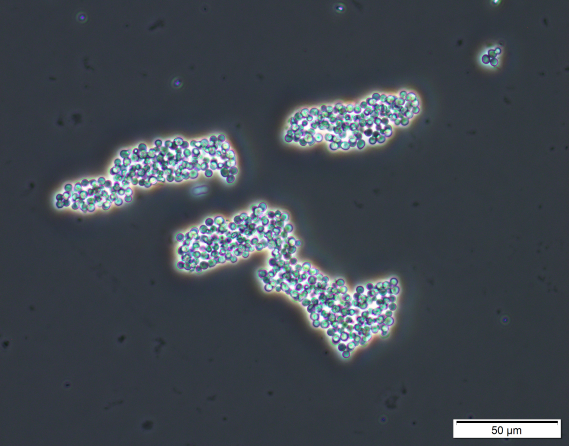

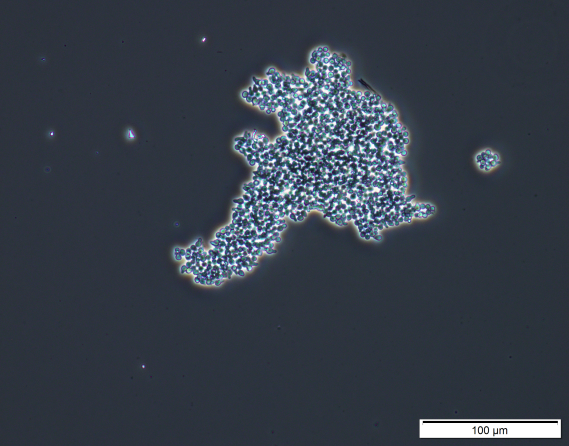

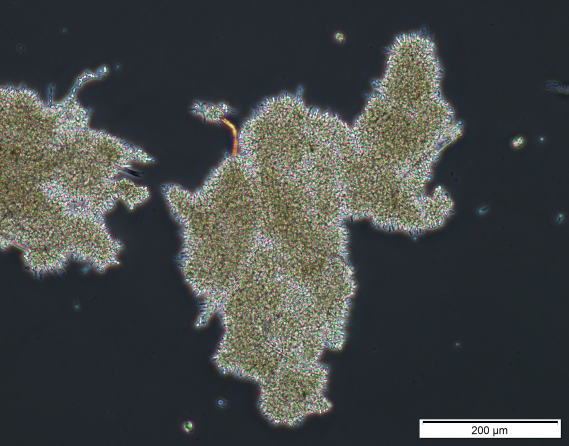


**h**

**g**

**i**


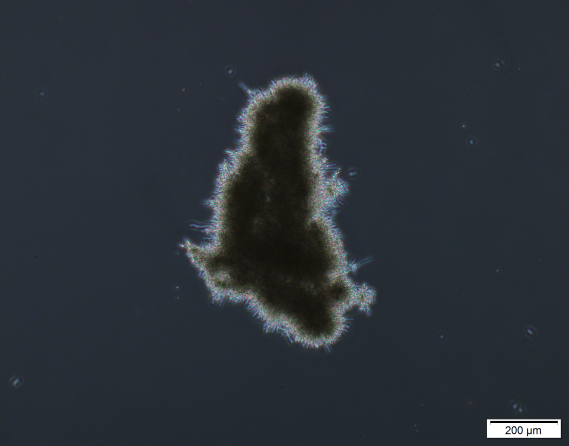

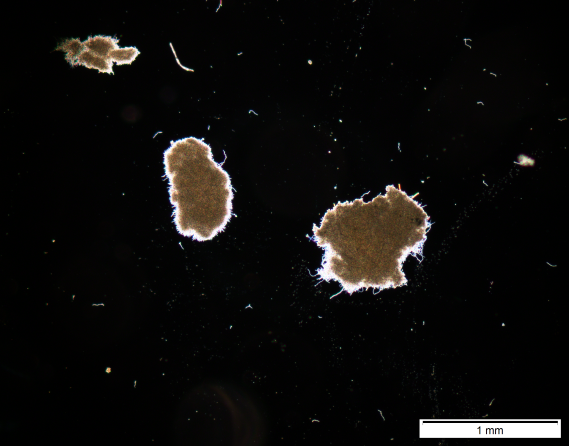

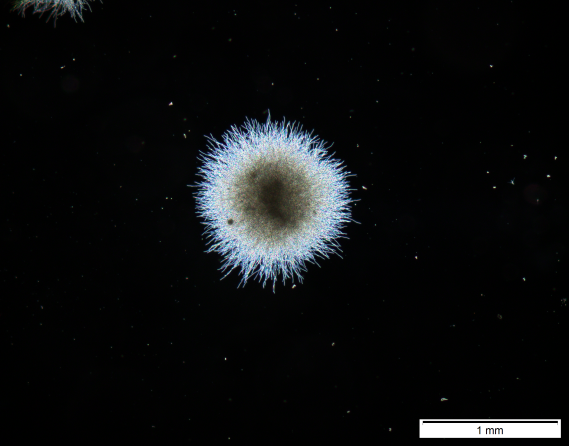


**j**


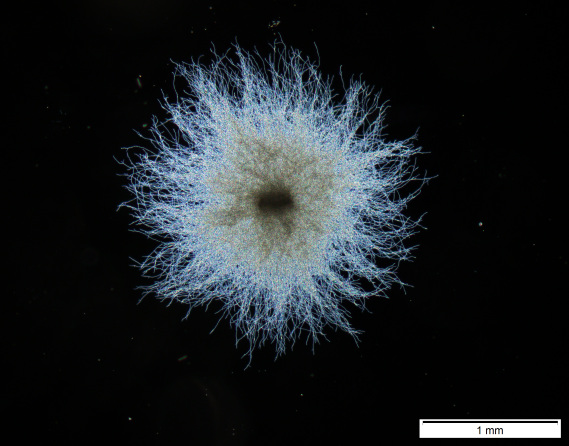


**Supplementary Fig. 3.** Microscopic images illustrating the mycelial development of *Aspergillus terreus* in liquid culture. The images were snapped at the following time points of the culture: (a) 0 h, (b) 5 h, (c) 6 h, (d) 7 h, (e) 8 h, (f) 10 h, (g) 12 h, (h) 15 h, (i) 17 h, (j) 24 h


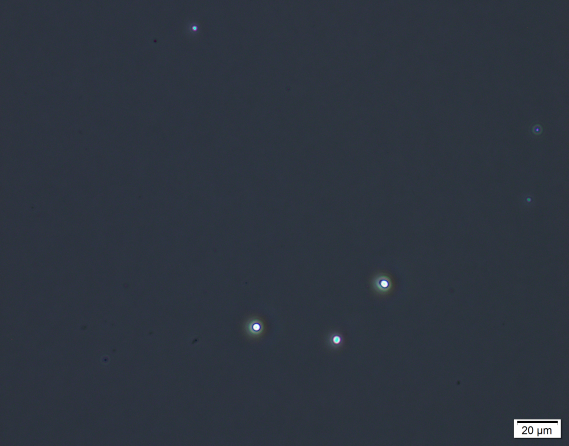

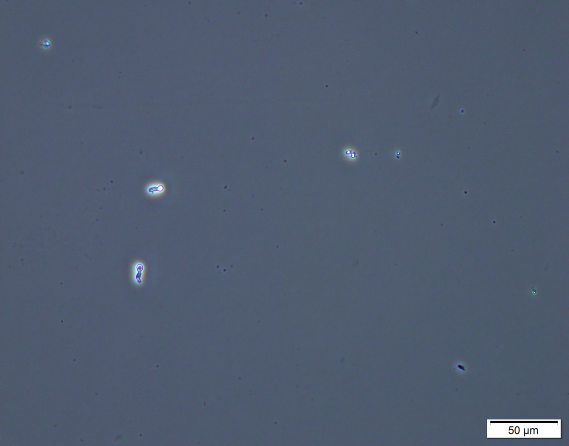

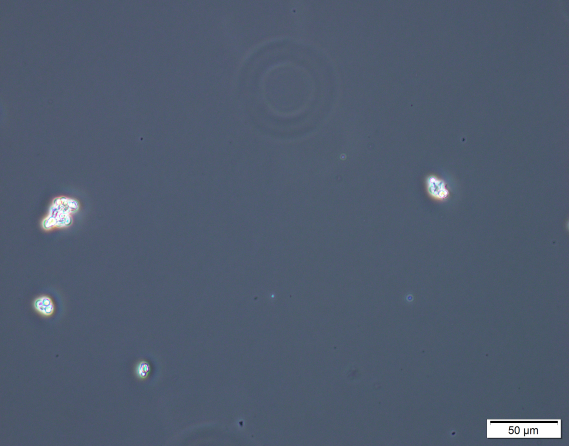


**c**

**b**

**a**

**d**

**e**

**f**

**g**

**h**


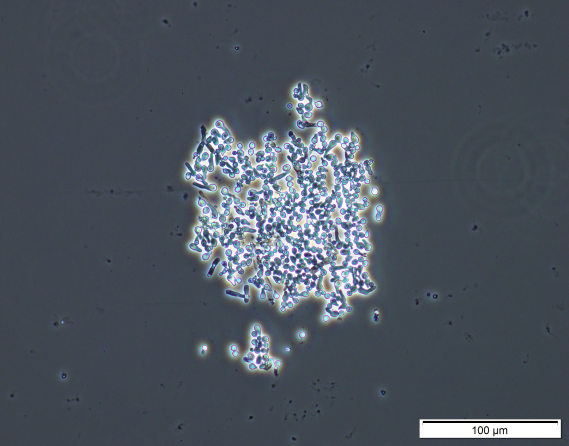

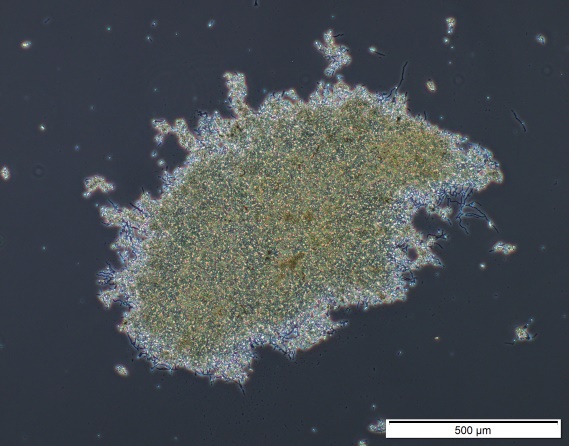

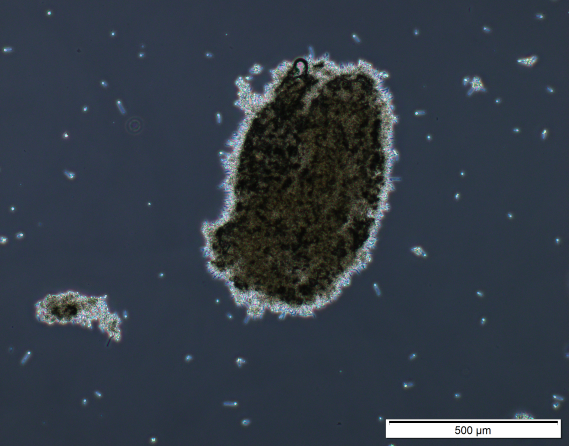


**i**


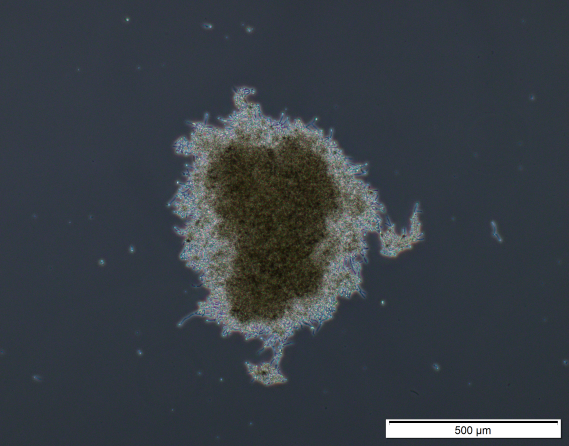

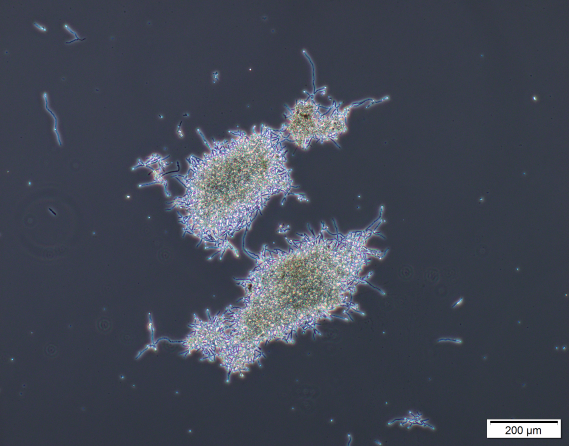

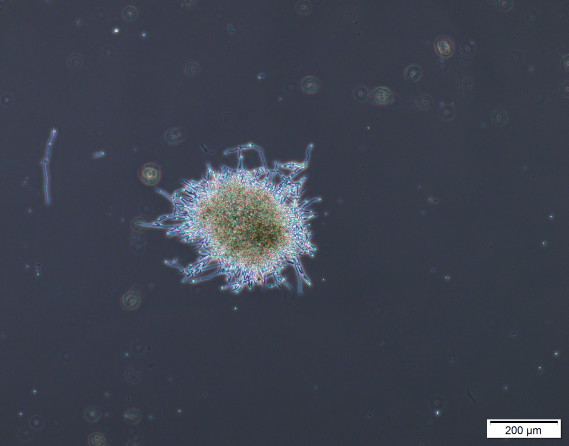


**j**


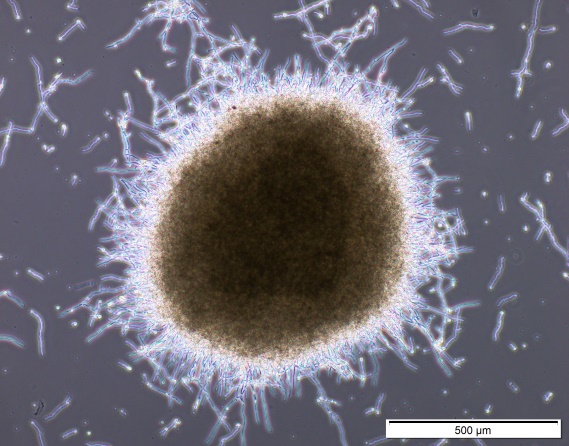


**Supplementary Fig. 4.** Microscopic images illustrating the mycelial development of *Penicillium rubens* in liquid culture. The images were snapped at the following time points of the culture: (a) 0 h, (b) 5 h, (c) 6 h, (d) 7 h, (e) 8 h, (f) 10 h, (g) 12 h, (h) 15 h, (i) 17 h, (j) 24 h


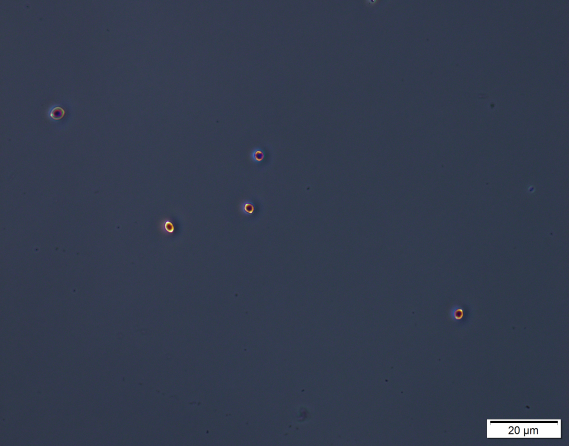

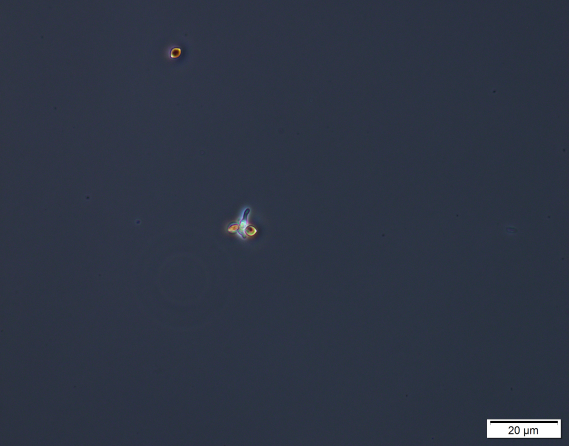

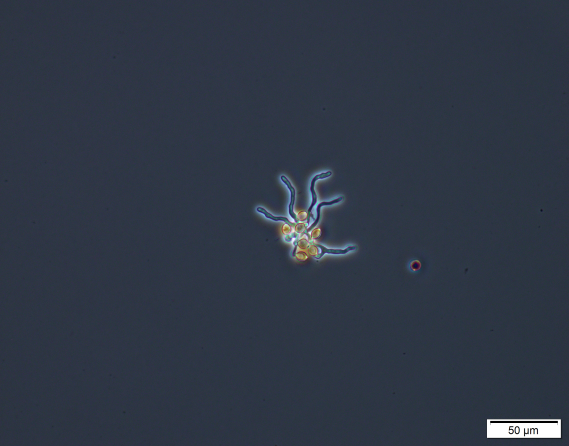


**c**

**b**

**a**

**d**

**e**

**f**

**g**

**i**

**h**

**j**


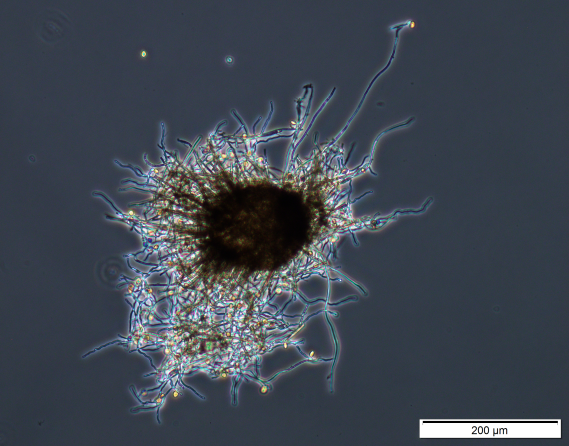

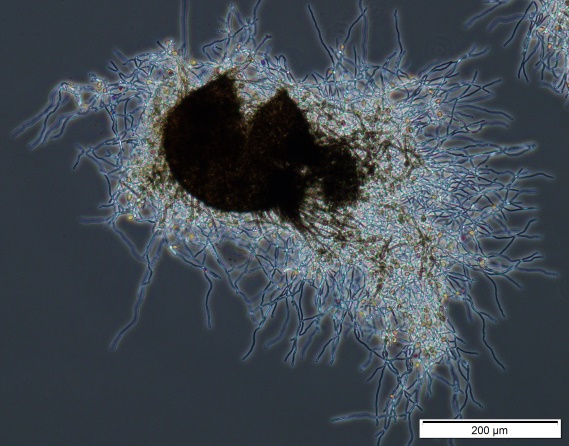

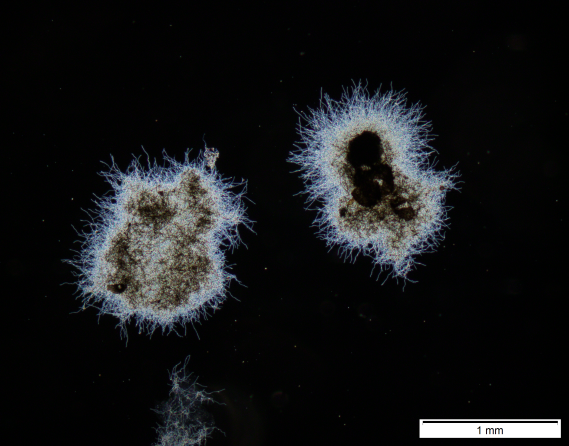


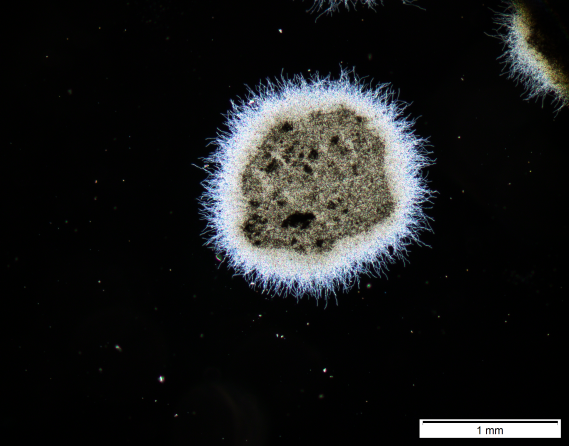

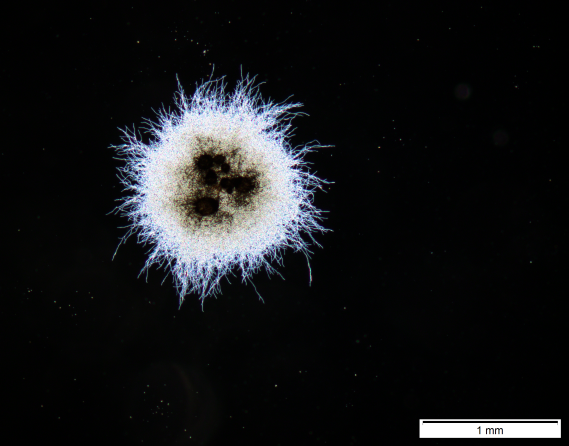

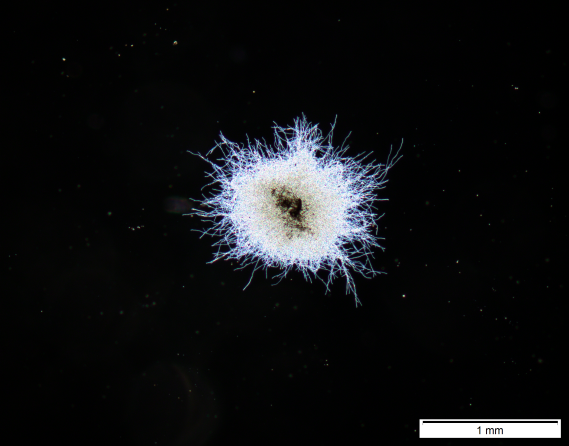


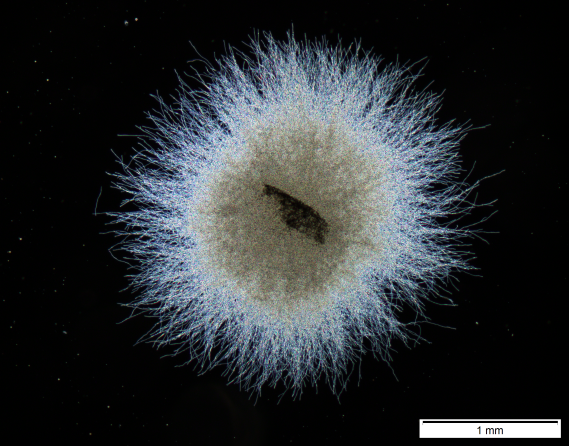


**Supplementary Fig. 5.** Microscopic images illustrating the mycelial development of *Chaetomium globosum* in liquid culture. The images were snapped at the following time points of the culture: (a) 0 h, (b) 5 h, (c) 6.5 h, (d) 8 h, (e) 10 h, (f) 12 h, (g) 14 h, (h) 16 h, (i) 18 h, (j) 24 h


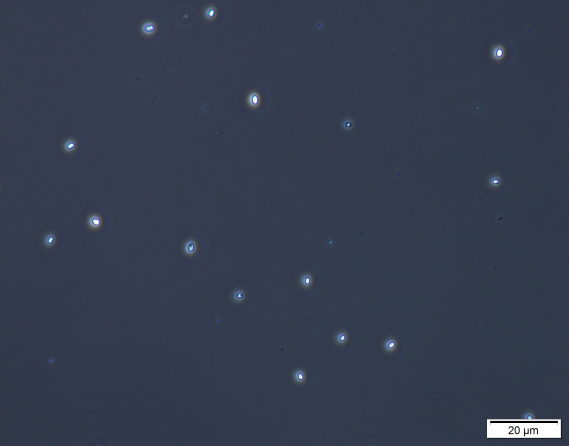

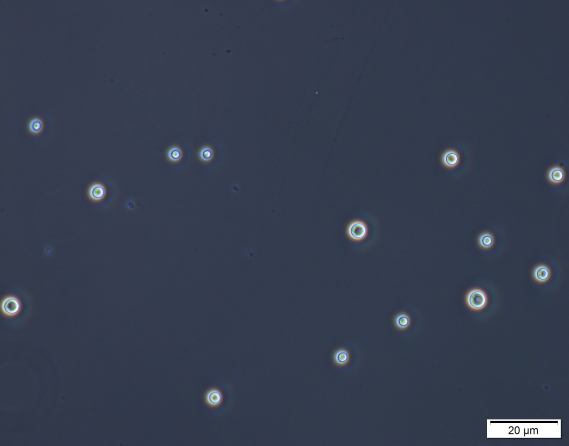

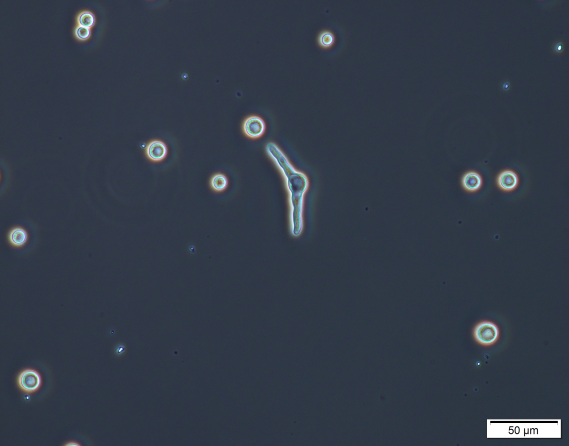


**c**

**b**

**a**

**d**

**e**

**f**

**g**

**i**

**h**

**j**


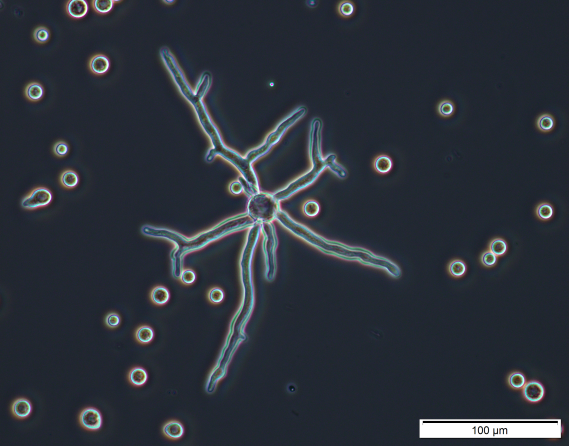

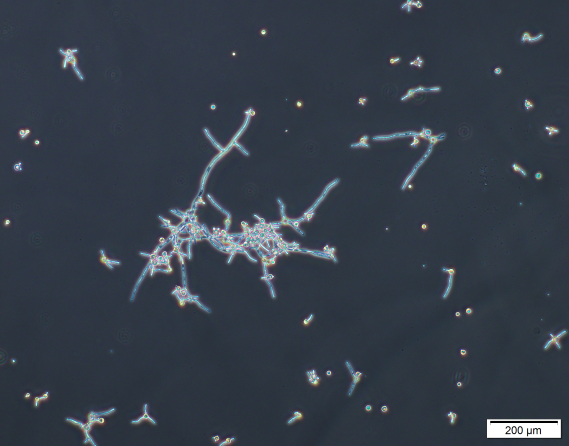

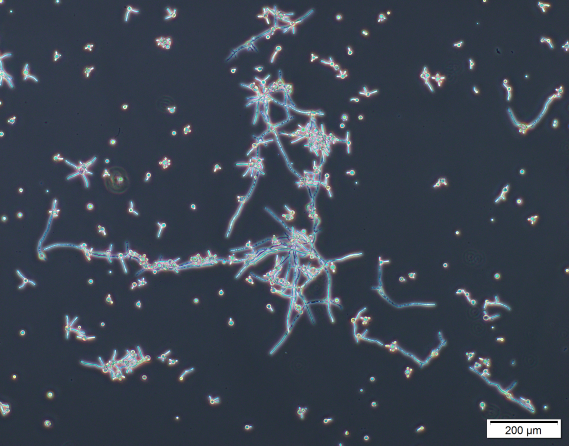


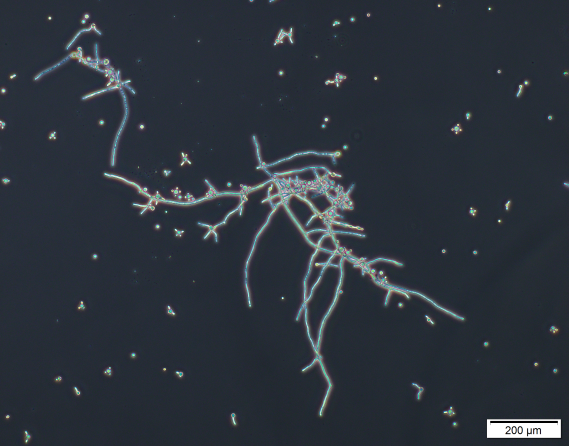

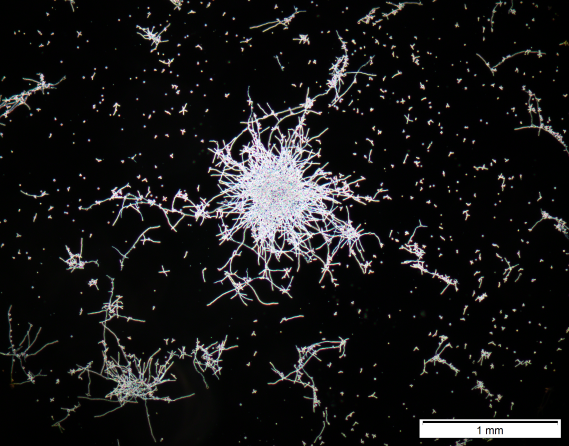

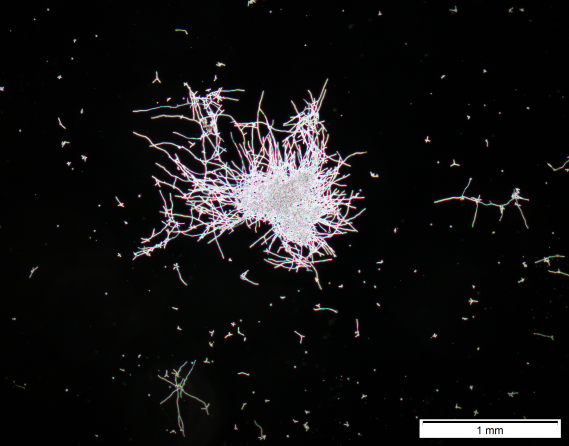


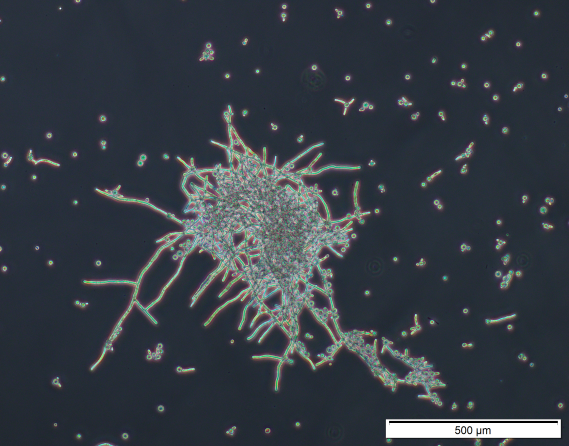


**Supplementary Fig. 6.** Microscopic images illustrating the mycelial development of *Mucor racemosus* in liquid culture. The images were snapped at the following time points of the culture: (a) 0 h, (b) 5 h, (c) 6.5 h, (d) 8 h, (e) 10 h, (f) 12 h, (g) 14 h, (h) 16 h, (i) 18 h, (j) 24 h


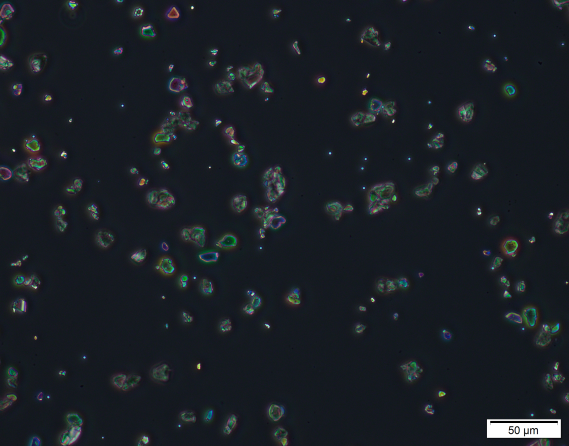

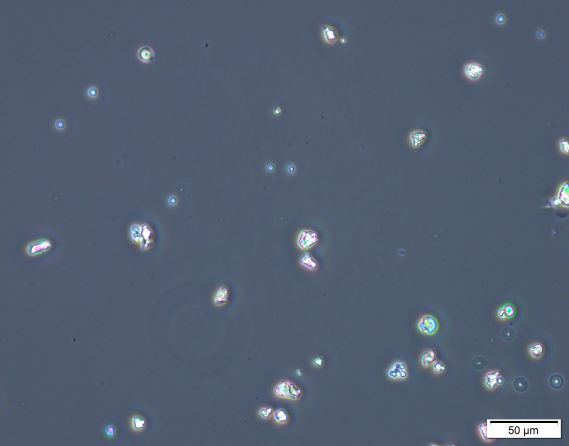

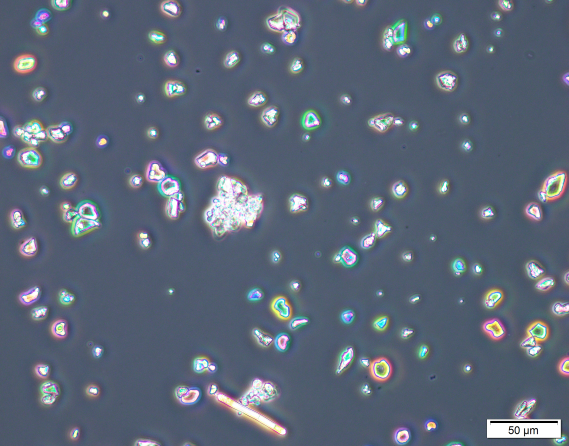


**j**

**h**

**i**

**g**

**f**

**e**

**d**

**a**

**b**

**c**


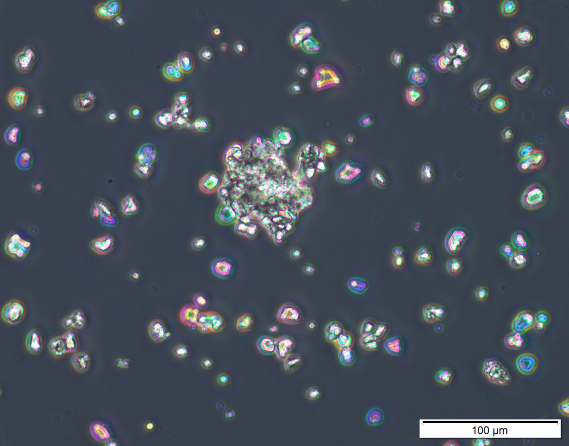

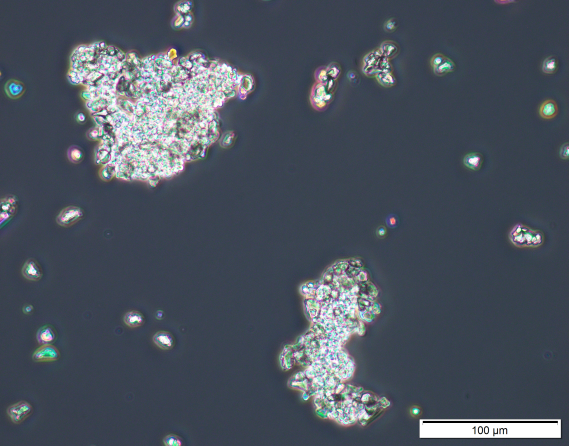

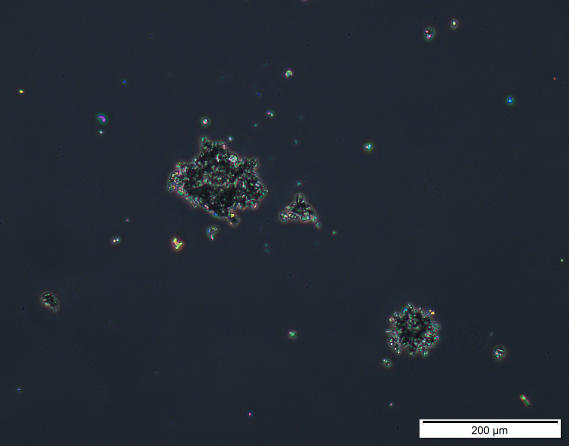


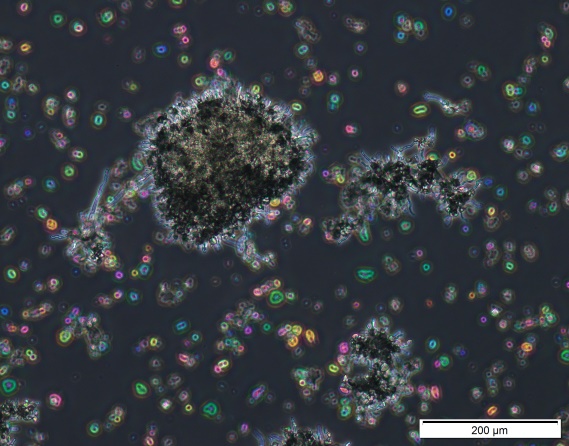

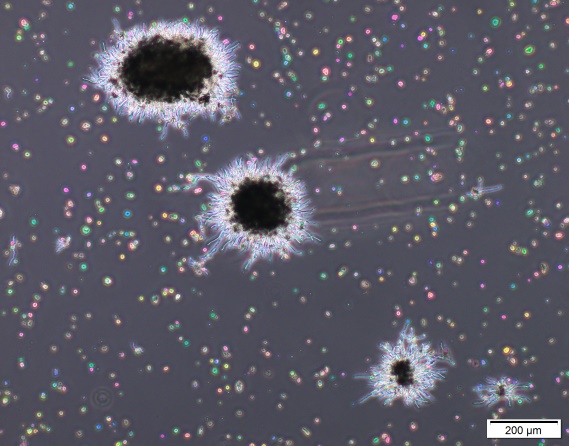

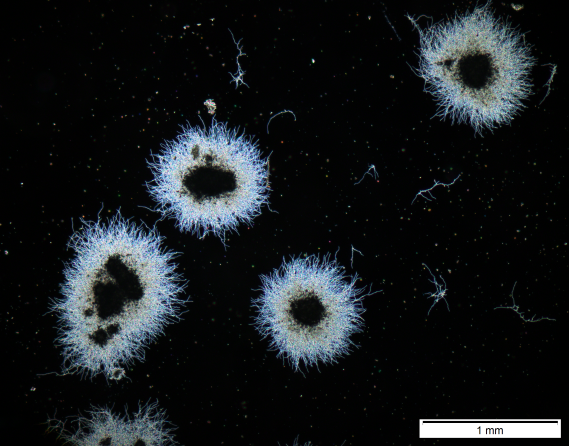


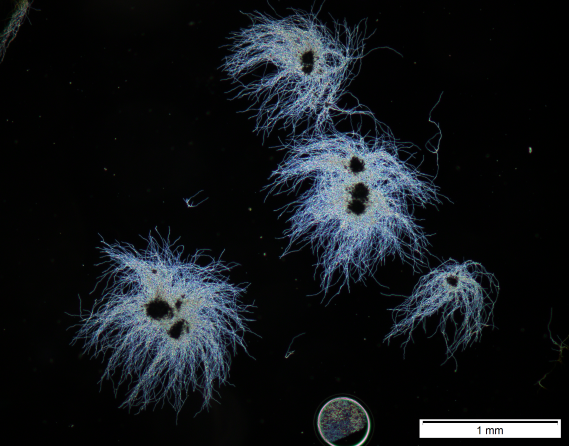


**Supplementary Fig. 7.** Microscopic images illustrating the mycelial development of *Aspergillus terreus* in the course of microparticle-enhanced cultivation (MPEC). The images were snapped at the following time points of the culture: (a) 0 h, (b) 5 h, (c) 6 h, (d) 7 h, (e) 8 h, (f) 10 h, (g) 12 h, (h) 15 h, (i) 17 h, (j) 24 h. Objects of particular interest are indicated by arrows.


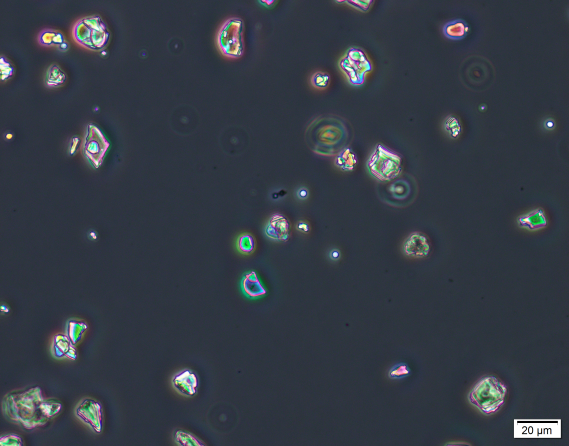

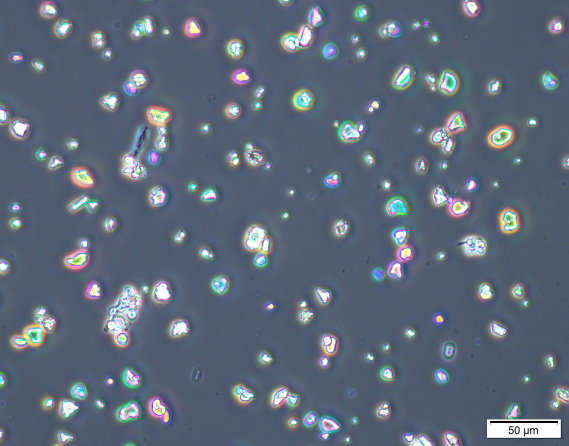

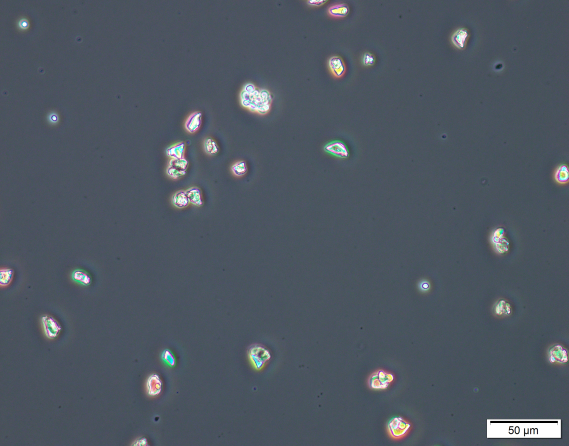


**j**

**h**

**i**

**g**

**f**

**e**

**d**

**a**

**b**

**c**


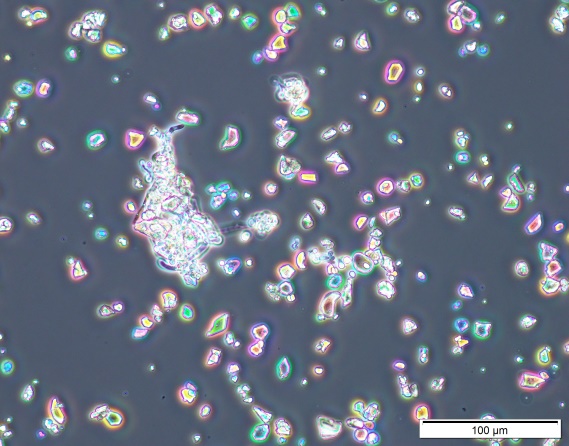

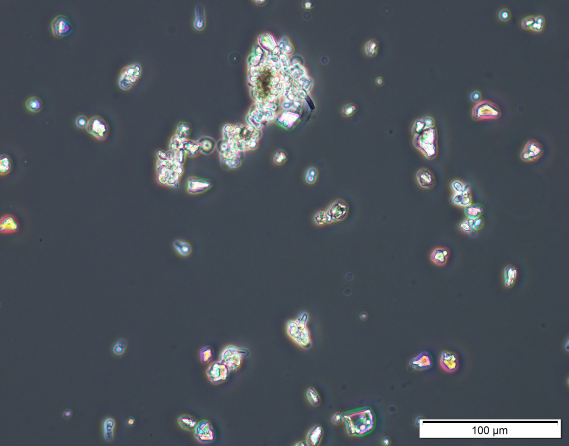

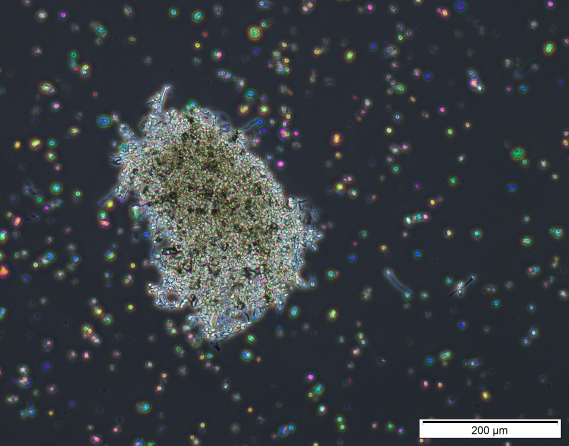


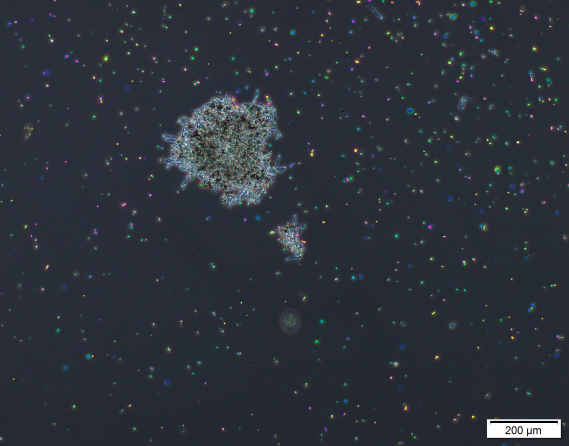

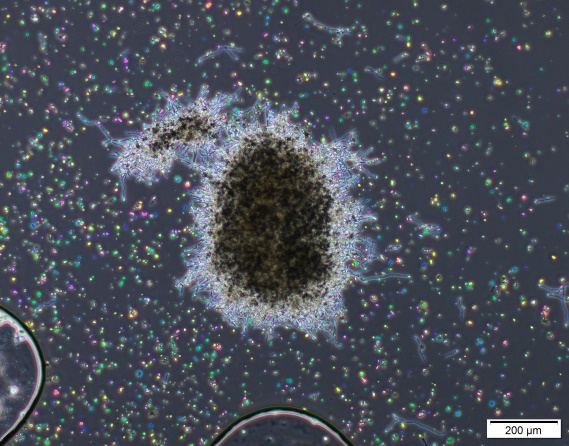

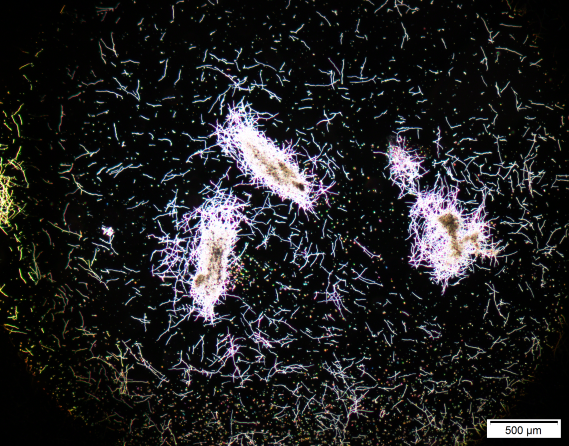


**Supplementary Fig. 8.** Microscopic images illustrating the mycelial development of *Penicillium rubens* in the course of microparticle-enhanced cultivation (MPEC). The images were snapped at the following time points of the culture: (a) 0 h, (b) 5 h, (c) 6 h, (d) 7 h, (e) 8 h, (f) 10 h, (g) 12 h, (h) 15 h, (i) 24 h. Objects of particular interest are indicated by arrows.


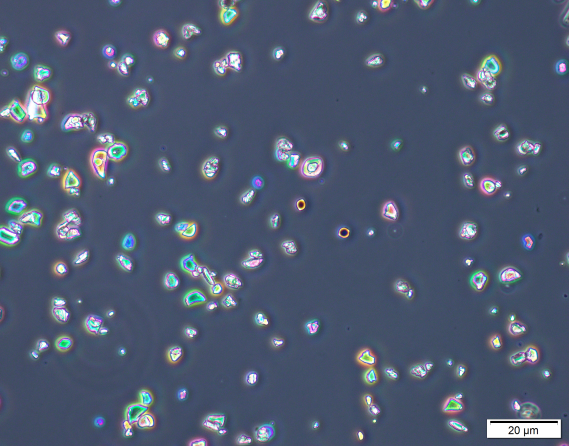

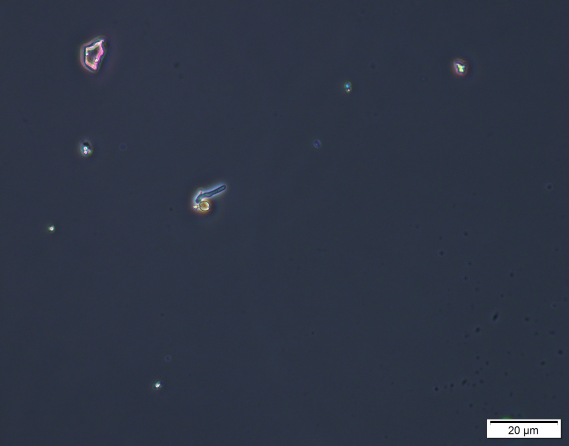

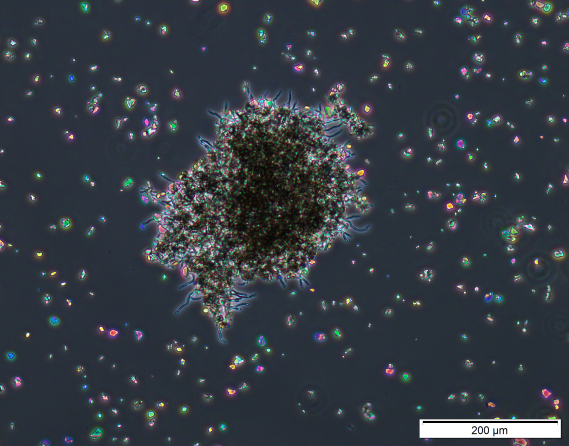


**j**

**h**

**i**

**g**

**f**

**e**

**d**

**a**

**b**

**c**


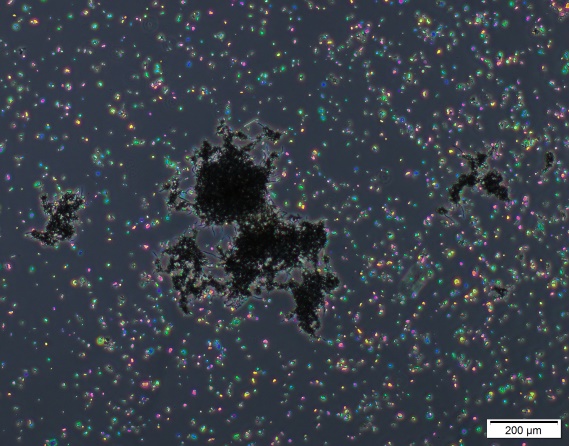

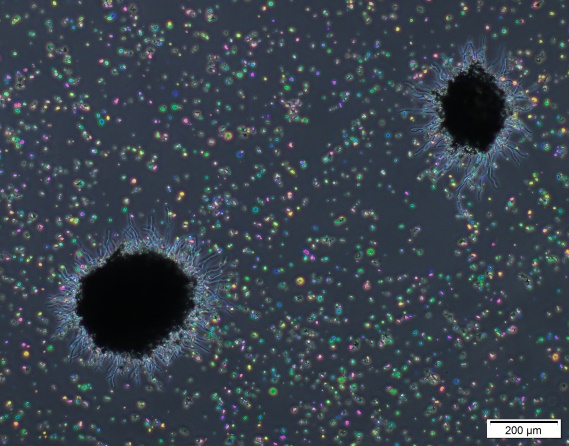

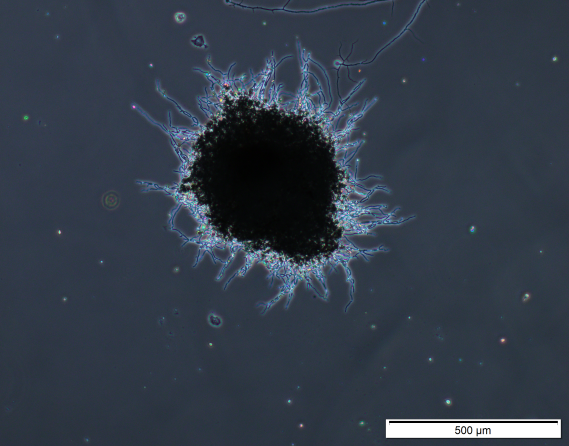


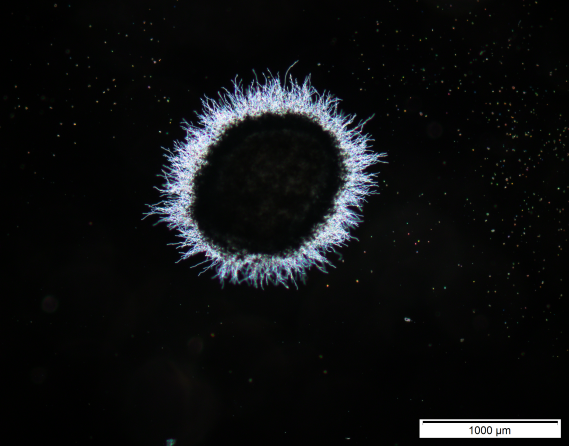

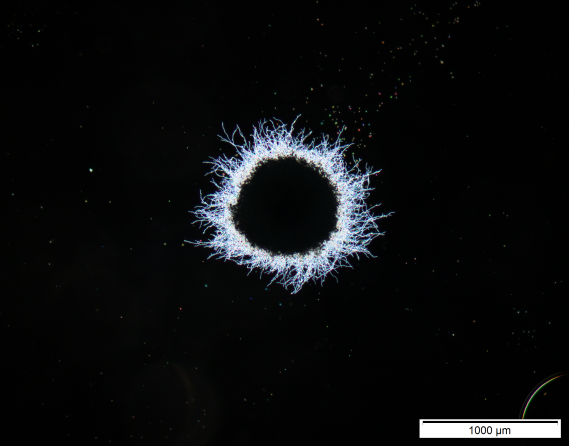

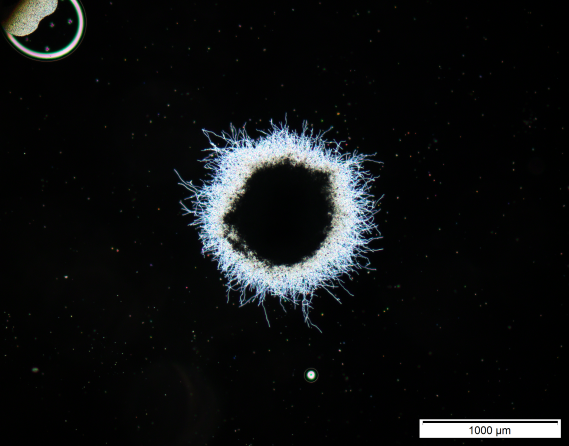


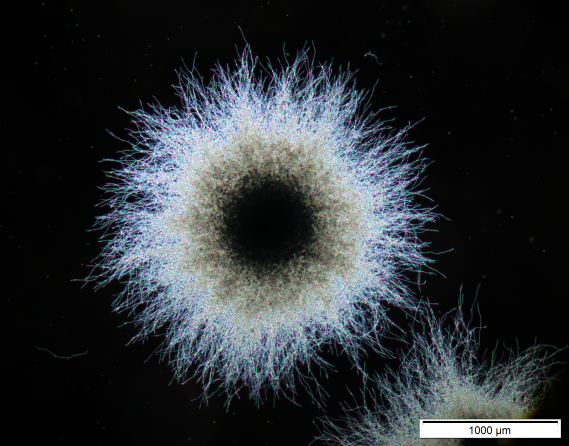


**Supplementary Fig. 9.** Microscopic images illustrating the mycelial development of *Chaetomium globosum* in the course of microparticle-enhanced cultivation (MPEC). The images were snapped at the following time points of the culture: (a) 0 h, (b) 5 h, (c) 6.5 h, (d) 8 h, (e) 10 h, (f) 12 h, (g) 14 h, (h) 16 h, (i) 18 h, (j) 24 h. Objects of particular interest are indicated by arrows.


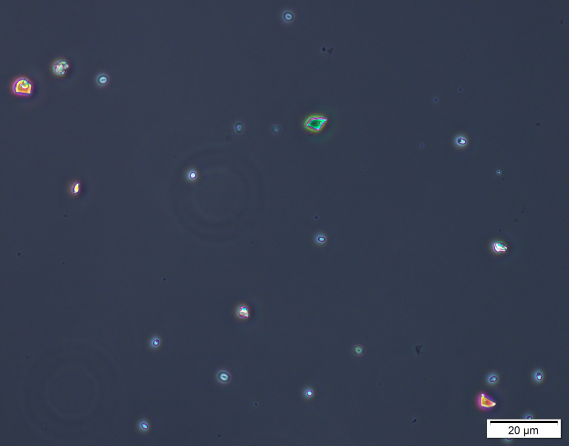

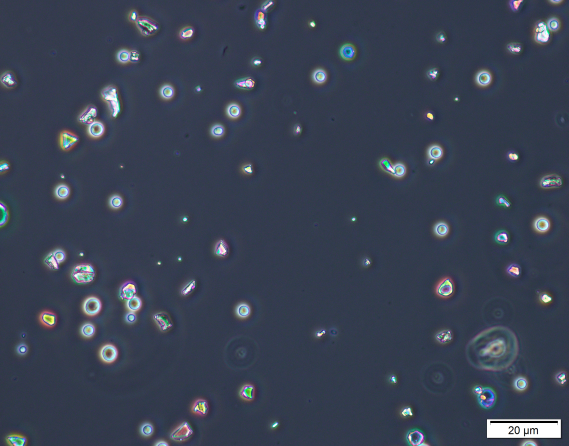

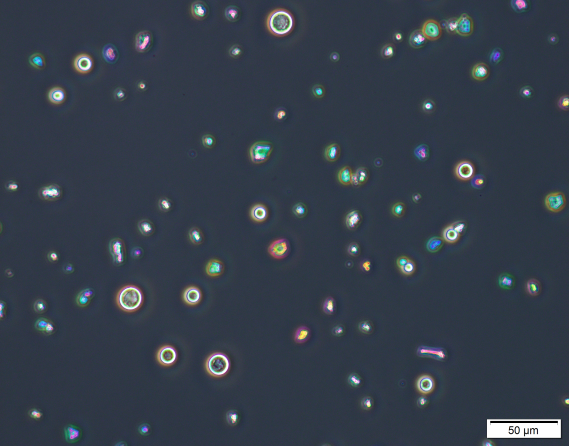


**j**

**h**

**i**

**g**

**f**

**e**

**d**

**a**

**b**

**c**


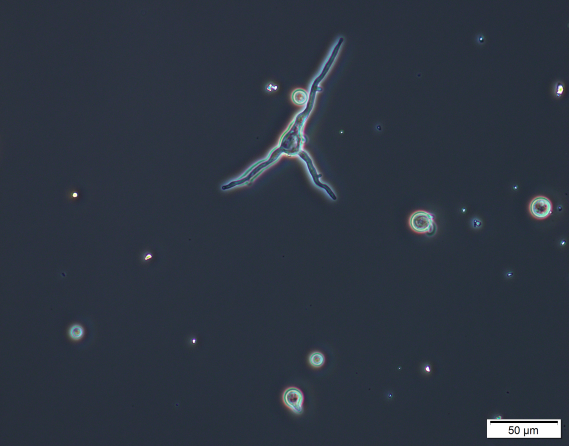

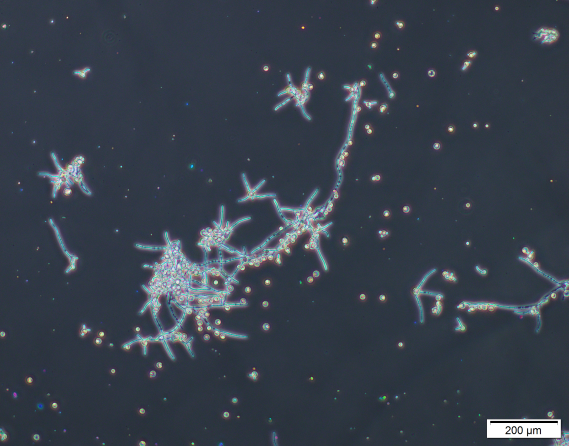

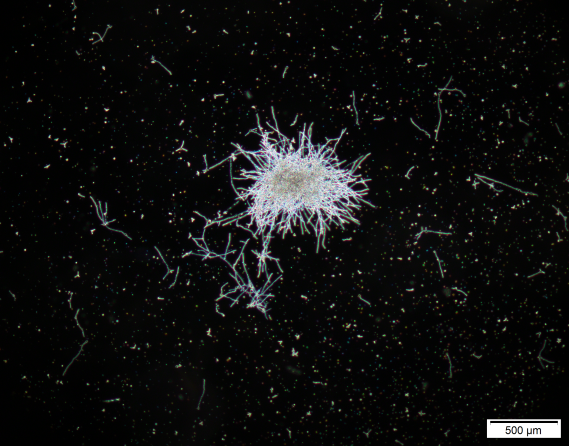


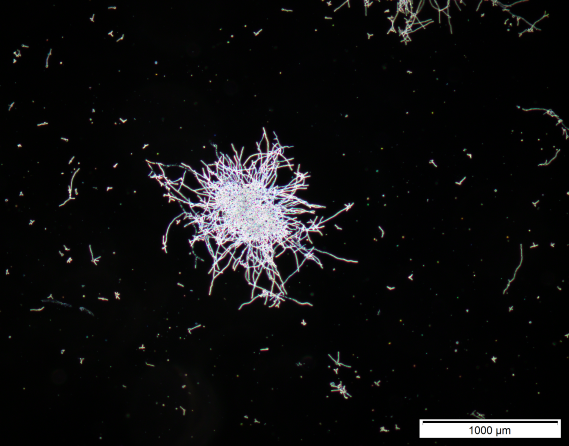

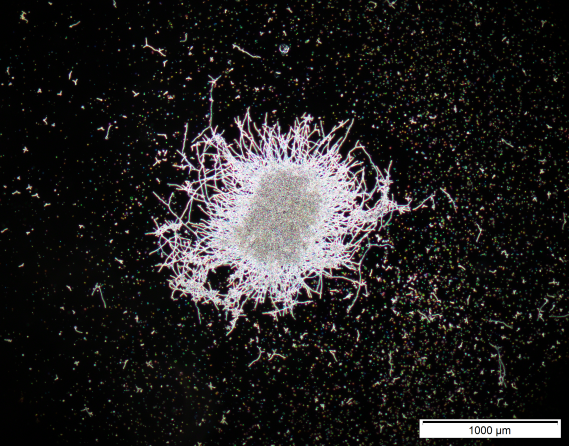

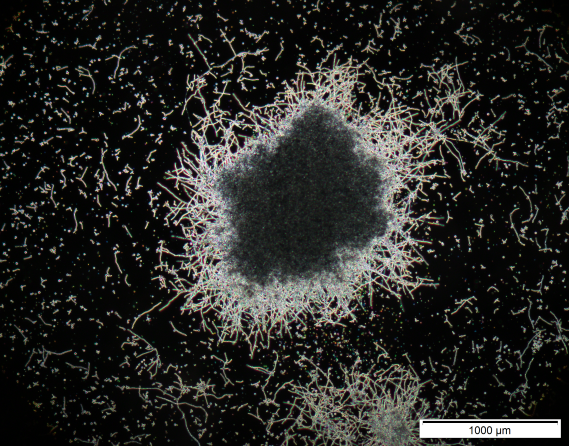


**Supplementary Fig. 10.** Microscopic images illustrating the mycelial development of *Mucor racemosus* in the course of microparticle-enhanced cultivation (MPEC). The images were snapped at the following time points of the culture: (a) 0 h, (b) 5 h, (c) 6.5 h, (d) 8 h, (e) 10 h, (f) 12 h, (g) 14 h, (h) 16 h, (i) 24 h. Objects of particular interest are indicated by arrows.
